# The multiple composite risk index estimates the risks of sarcopenia and comorbidities: unveiling the role of oxidative balance score

**DOI:** 10.1080/07853890.2026.2723681

**Published:** 2026-09-02

**Authors:** Minghao Liu, Xiaohu Chu, Ding Zeng, Shuaifei Ji, Xin Miao

**Affiliations:** a Department of Vascular Surgery, The First Medical Centre, Chinese PLA General Hospital, Beijing, China; b PLA Rocket Force Characteristic Medical Center, Beijing, China; c Senior Department of General Surgery, Chinese PLA General Hospital, Beijing, China

**Keywords:** Purine metabolism, hyperuricemia, lipid metabolism, obesity, oxidative stress, sarcopenia

## Abstract

**Background:**

Obesity, purine metabolism disorders, and dyslipidemia are recognized risk factors for sarcopenia, yet reliable biomarkers for risk stratification in this population are lacking. We integrated the uric acid to high-density lipoprotein cholesterol ratio (UHR) with the body roundness index (BRI) to construct a multiple composite risk index, UHR-BRI, and evaluated its associations with sarcopenia and its comorbidities.

**Methods:**

We analyzed two nationally representative cohorts: the China Health and Retirement Longitudinal Study (CHARLS) and the National Health and Nutrition Examination Survey (NHANES). The association between UHR-BRI and sarcopenia was evaluated using weighted logistic regression. Subgroup and mediation analyses were performed to investigate the interactions across subgroups, and the mediating role of oxidative balance score (OBS).

**Results:**

A total of 8,537 participants from CHARLS and 8,510 from NHANES were included, among whom 1,082 and 716 were diagnosed with sarcopenia. The area under the curve (AUC) value of the UHR-BRI index was higher than that of the other indices in both cohorts. In fully adjusted models, participants in the highest UHR-BRI quartile exhibited a significantly increased risk of sarcopenia (OR:3.01, 95% CI:2.45–3.72) compared to those in the lowest quartile in CHARLS. This finding was validated in NHANES (OR:17.14, 95% CI:11.55–26.60). Subgroup analyses further demonstrated robustness across different subgroups. Furthermore, UHR-BRI was also significantly associated with sarcopenia comorbidities. Mediation analyses confirmed that OBS mediated the associations between UHR-BRI and sarcopenia and its comorbidities.

**Conclusion:**

UHR-BRI is significantly associated with sarcopenia and its common comorbidities, and OBS was identified as a potential mediator.

## Introduction

Sarcopenia is a syndrome characterized by progressive and generalized decline in skeletal muscle mass, strength, and physical function [1,2]. It not only impairs physical performance and activities of daily living, markedly raising the risks of falls, disability, and all-cause mortality [3], but also frequently coexists with chronic diseases including obesity, diabetes, and cardiovascular disease [4,5]. These comorbidities accelerate muscle catabolism, fostering a vicious pathogenic cycle that severely impairs quality of life and impose a substantial burden on healthcare systems, thus creating an urgent need for effective risk stratification tools.

The development and progression of sarcopenia are driven by the combined effects of multiple pathogenic factors [6]. Dysregulated purine and lipid metabolism represent core metabolic drivers, and obesity is a well-established risk factor. These factors act synergistically to further amplify sarcopenia risk. Accordingly, developing a composite index integrating purine metabolism, lipid metabolism, and obesity status has substantial clinical value for early identification of sarcopenia in high-risk populations.

Purine metabolism is tightly linked to sarcopenia onset and progression [7,8]. As the terminal metabolite of the purine pathway, uric acid triggers mitochondrial damage in skeletal muscle by mediating oxidative stress, chronic low-grade inflammation, and insulin resistance, which in turn exacerbates sarcopenia [9]. Dyslipidemia is another key modifiable risk factor for sarcopenia, with reduced high-density lipoprotein cholesterol (HDL-C) levels strongly correlated with muscle mass loss and muscle weakness [10]. The uric acid to high-density lipoprotein cholesterol ratio (UHR) simultaneously reflects dual abnormalities in purine metabolism and lipid metabolism [11,12]. Compared with traditional indices reflecting hyperglycemia and hyperlipidemia, UHR better reflects the contribution of uric acid metabolism to sarcopenia progression, offering a novel metabolic marker for sarcopenia risk stratification and mechanistic investigation.

Obesity, particularly central obesity, is a major risk factor for sarcopenia [2,13]. Conventional anthropometric measures of adiposity, such as body mass index (BMI) and waist circumference (WC), have notable limitations in characterizing adipose tissue distribution. In recent years, the Body Roundness Index (BRI), based on geometric analysis of body shape with a focus on the relationship between abdominal fat distribution and height, has been widely recognized as a reliable indicator for evaluating central obesity. Multiple studies have demonstrated that elevated BRI is strongly associated with higher sarcopenia risk [14,15].

To address this research gap regarding the combined effects of purine metabolism and lipid metabolism on sarcopenia in obese populations, this study developed a composite index termed UHR‑BRI. This index integrates UHR and BRI, focusing on the joint effects of purine metabolism, lipid metabolism, and abdominal obesity, and is particularly suitable for sarcopenia risk screening in high‑risk populations such as individuals with obesity and hyperuricemia. Using data from two nationally representative cohorts, the China Health and Retirement Longitudinal Study (CHARLS) and the National Health and Nutrition Examination Survey (NHANES), this study aimed to investigate the associations of UHR‑BRI with sarcopenia and its comorbidities, and to further explore the potential mediating role of oxidative balance score (OBS).

## Methods

### Study design and population

This study utilized data from two nationally representative cohorts: the CHARLS and the NHANES. CHARLS is a nationally representative longitudinal survey led by Peking University, enrolling Chinese residents aged 45 years and older across urban and rural regions. Between 2011 and 2012 (Wave 1), a total of 17,708 participants from 150 counties across 28 provinces were recruited nationwide, with follow-up surveys conducted in 2013, 2015, 2018, and 2020 (Wave2-4) [16]. NHANES, conducted by the National Center for Health Statistics (NCHS), is a nationally representative cross-sectional survey designed to systematically assess the health and nutritional status of the U.S. population [17]. Both surveys adopted multistage probability sampling designs and collected data across multiple domains, including demographic characteristics, physical examination indicators, biomarkers, socioeconomic status, and lifestyle factors. The study protocols were approved by the respective ethics review boards, and all participants provided written informed consent. This study utilized data from CHARLS Waves 1–5 (2011–2020) and NHANES cycles 2011–2018. Data collection and analysis were performed between February 2026 and May 2026.

In the CHARLS cohort, we excluded participants with missing sarcopenia diagnosis data (*n* = 7,322), missing values for UHR and BRI calculation (*n* = 762), as well as those with missing follow-up information (*n* = 390) or other baseline information (*n* = 697), a total of 8,537 participants were ultimately included. In the NHANES cohort, we utilized data from 2011 to 2018. A total of 39,156 participants were initially enrolled. After excluding individuals aged <20 years (*n* = 7,084), those missing information for sarcopenia diagnosis (*n* = 21,230), those with missing data on UHR or BRI (*n* = 1,326), and those lacking other key baseline information (*n* = 1,006), a final sample of 8,510 participants was included for analysis. The flowchart of this study is shown in Figure S1.

### The definitions of UHR-BRI and other indices

Serum levels of uric acid, total cholesterol (TC), triglycerides (TG), fasting blood glucose (FBG) and HDL-C were measured from fasting blood samples of participants. The UHR was calculated as uric acid (mg/dL) divided by HDL-C (mg/dL). Furthermore, information on height, body weight, and waist circumference was collected using standardized measurement tools, and a series of obesity-related parameters were calculated, including BMI, WC, BRI, A Body Shape Index (ABSI), Waist-to-Height Ratio (WHtR), and Weight-adjusted Waist Index (WWI). UHR-BRI was constructed as the product of UHR and BRI, because UHR and BRI reflect distinct metabolic dimensions and it follows the construction principle of established composite indices, such as the TyG-derived indices (TyG-BMI, TyG-WC). This approach yields a simple, reproducible, and cohort-independent measure. The specific calculation formulas are as follows:

$$\begin{array}{l} \text{UHR}-\text{BMI}=\text{UHR}\times \text{BMI}\\ \text{UHR}-\text{WC}=\text{UHR}\times \text{WC}\\ \text{UHR}-\text{WHtR}=\text{UHR }\times \text{ WC}/\text{height}\\ \text{UHR}-\text{ABSI=UHR }\times \text{ WC/[BMI\textasciicircum{}(2/3) }\times \text{ height\textasciicircum{}(1/2)]}\\ \text{UHR}-\text{WWI=UHR }\times \text{ height/[weight\textasciicircum{}(1/2)]}\\ \text{UHR}-\text{BRI=UHR }\times \;\left[364\text{.2 }-\text{ 365.5 }\times \;\surd \left(1-{\left(\text{WC/2}\pi \right)}^{2}\text{/}{\left[0\text{.5 }\times \text{ height}\right]}^{2}\right)\right]\\ \text{AIP}=\text{log1}0\left(\text{TG}/\text{HDL}-C\right)\\ \text{TyG}=\text{ln}\left[\text{TG}\times \text{FBG}/2\right]\\ \text{CHG}=\text{ln}\left[\text{TC}\times \text{FBG}/\left(2\times \text{HDL}-C\right)\right] \end{array}$$

Furthermore, we calculated the cumulative UHR-BRI index (cuUHR-BRI) by averaging the UHR-BRI values from 2012 and 2015 and multiplying by the time interval between assessments: (UHR-BRI wave1 +UHR-BRI wave3)/2 × time interval (2015 − 2012).

### Definition of sarcopenia

In the NHANES cohort, appendicular skeletal muscle mass (ASM), defined as the sum of muscle mass in the four limbs, was precisely measured using dual-energy X-ray absorptiometry (DXA). Relative muscle loss was used as a proxy for sarcopenia. According to the consensus criteria of the Foundation for the National Institutes of Health (FNIH) [18], muscle loss was defined as an ASM/BMI ratio of <0.789 for men and <0.512 for women. In the CHARLS cohort, based on the 2019 Asian Working Group for Sarcopenia (AWGS 2019) consensus [19], sarcopenia was defined as a gradual decline in muscle mass, muscle strength, and/or physical capability, and its assessment incorporated three core components: ASM, muscle strength, and physical performance; ASM was estimated using a validated equation: ASM = 0.193 × weight (kg) + 0.107 × height (cm) − 4.157 × sex (male = 1, female = 2) − 0.037 × age − 2.631, and was normalized by height squared (ASM/Ht^2^, kg/m^2^) to determine low muscle mass. The threshold was established at the 20th percentile, corresponding to values below 7.03 kg/m^2^ for males and 5.39 kg/m^2^ for females. Muscle strength was assessed by a dynamometer, with low muscle strength defined as handgrip strength <28 kg for males and <18 kg for females; physical performance was evaluated by chair stand test and walking speed, with low physical performance defined as a time of ≥12 s to complete the chair stand test or a walking pace of less than 1.0 m per second [20].

### Definition of OBS

In the NHANES cohort, we calculated the OBS, a comprehensive indicator for assessing individual oxidative stress status. The OBS was derived by integrating 16 dietary nutrients and 4 lifestyle factors, reflecting the total oxidative stress burden of the body [21]. Intake of each nutrient was calculated as the average of two 24-hour dietary recalls. Physical activity data were used to calculate total weekly metabolic equivalent minutes (MET-min/week) based on activity type, frequency, and duration. In accordance with the 2018 Physical Activity Guidelines for Americans [22], participants were categorized into highly active (≥1200 MET-min/week), active (600–1200 MET-min/week), and inactive (<600 MET-min/week) groups. This analysis was not performed in the CHARLS cohort due to the lack of specific nutritional indicators.

### Covariates

The following covariates were collected: (1) sociodemographic factors: age, sex, race/ethnicity, poverty income ratio (PIR), education level, and marital status; (2) lifestyle factors: smoking and alcohol consumption status. Smoking status was categorized as non-smoker (<100 cigarettes in lifetime) or smoker (≥100 cigarettes). Alcohol consumption was defined as non-drinker (<12 alcoholic drinks/year) or drinker (≥12 drinks/year); (3) laboratory examinations: alanine aminotransferase (ALT), aspartate aminotransferase (AST), creatinine (Cr) and so on. For covariates with missing data (below 20%), multiple imputation by chained equations (MICE) was applied to ensure the robustness of the analyses. Detailed imputation procedures are presented in the Supplementary Materials.

### Statistical analysis

Descriptive statistics were used to summarize the baseline characteristics of the participants. Continuous variables were presented as mean ± standard deviation (SD), and comparisons between groups were performed using one-way analysis of variance (ANOVA). Categorical variables were expressed as frequencies and percentages, and group differences were assessed using the chi-square test.

Several logistic regression models were constructed to estimate odds ratios (ORs) with corresponding 95% confidence intervals (CIs): (1) Model 1: unadjusted; (2) Model 2: adjusted for age and sex; (3) Model 3: further adjusted for marital status, educational level, TG, FBG, Cr, Blood Urea Nitrogen (BUN), smoking, and alcohol use. To assess the joint effect of UHR and BRI on sarcopenia outcomes, we included the product of UHR and BRI in the models to test for multiplicative interaction. Additive interaction was further evaluated using the relative excess risk due to interaction (RERI), attributable proportion (AP), and synergy index (SI).

Receiver operating characteristic (ROC) curves were used to evaluate and compare the area under the curve (AUC) value of the UHR-BRI with that of other indices, including UHR, BRI, UHR-based indices, and other established metabolic indices (e.g. CHG, TyG, AIP). In the CHARLS cohort, we additionally performed trajectory analysis using Group-Based Trajectory Modeling (GBTM) with a finite mixture linear model to characterize the longitudinal change patterns of UHR‑BRI from Wave 1 to Wave 3. Subsequently, the Boruta algorithm and logistic regression analysis were employed to select variables, and the intersection of the results was defined as independent risk factors. Based on these factors, an XGBoost machine learning model was constructed, and Shapley additive explanations (SHAP) were used to quantify and visualize the importance of each feature in the model [23].

We then identified participants with comorbidities of sarcopenia and obesity, diabetes, kidney disease, cancer, stroke, or cardiovascular disease (CVD). Diabetes was defined as meeting at least one of the following criteria: (1) fasting plasma glucose ≥126 mg/dL or 2-h oral glucose tolerance test ≥200 mg/dL; (2) random plasma glucose ≥200 mg/dL; (3) glycated hemoglobin (HbA1c) ≥6.5%; (4) current use of antidiabetic medication or insulin; or (5) self-reported physician diagnosis. The diagnosis of other medical conditions was ascertained by self-report. The association between UHR-BRI and sarcopenia with comorbidities was analyzed using the previously constructed logistic regression models. Restricted cubic spline (RCS) analyses were further employed to explore nonlinear associations. In addition, subgroup analyses were performed to examine interactions with covariates, and p-values for interaction were calculated. Mediation analysis was conducted to explore the mediating role of OBS in the relationship between UHR-BRI and sarcopenia and its comorbidities. Finally, to further ensure the robustness of the findings, sensitivity analyses were performed by replicating the main analyses using the complete-case dataset without imputation. All analyses in this study were conducted using R software (version 4.5.3), and two-sided p-values <0.05 were considered statistically significant.

## Results

### Baseline characteristics

According to the inclusion and exclusion criteria, a total of 8,537 eligible participants were enrolled in the CHARLS cohort, with a mean age of 59.5 years, of whom 4,534 (53.1%) were male. Sarcopenia was identified in 1,082 participants, yielding an overall prevalence of 12.7%. In the NHANES cohort, 8,510 participants were included (mean age: 39.1 years; male: 49.7%), among whom 716 were diagnosed with sarcopenia (8.4%). After stratifying participants into eight subgroups based on the median of UHR and quartiles of BRI, the baseline characteristics are presented in Table S1 and S2. In both cohorts, participants in Type 8 were older, the mean age increased from 58.9 years in Type 1 to 60.8 years in Type 8 in CHARLS, and from 34.3 years to 40.8 years in NHANES. Levels of biochemical markers including TC, TG, FBG, and BUN were also significantly elevated in Type 8, along with higher proportions of smoking and alcohol consumption. In the CHARLS cohort, 303 participants (23.5%) in Type 8 had sarcopenia, which was substantially higher than that in Type 1 (3.1%). Similarly, in NHANES, sarcopenia was 20.9% in Type 8 (vs. 0.7% in Type 1), and all differences were statistically significant. The baseline characteristics of participants stratified by sarcopenia status are shown in Table S4 and S5.

**Table 1.** Baseline characteristics of the participants according to the UHR-BRI quartiles in the CHARLS cohort.

Variables	Overall	Q1	Q2	Q3	Q4	P-value
	(*N* = 8537)	(*N* = 2144)	(*N* = 2129)	(*N* = 2137)	(*N* = 2127)	
Age, years, mean (SD)	59.5 (9.3)	58.9 (9.3)	59.1 (9.1)	59.4 (9.5)	60.6 (9.4)	<0.001
Gender, n (%)						0.011
Male	4,534 (53.1%)	1148(53.5%)	1100(51.7%)	1194(55.9%)	1092(51.3%)	
Female	4,003 (46.9%)	996(46.5%)	1029(48.3%)	943(44.1%)	1035(48.7%)	
Marital Status, n (%)						0.311
Single	1040 (12.2%)	281(13.1%)	250(11.7%)	267(12.5%)	242(11.4%)	
Married	7497 (87.8%)	1863(86.9%)	1879(88.3%)	1870(87.5%)	1885(88.6%)	
Educational Level, n (%)						<0.001
Less than high school	5975 (70.0%)	1548(72.2%)	1505(70.7%)	1488(69.6%)	1434(67.4%)	
High school	2295 (26.9%)	546(25.5%)	580(27.2%)	568(26.6%)	601(28.3%)	
College or above	267 (3.1%)	50(2.3%)	44(2.1%)	81(3.8%)	92(4.3%)	
Cholesterol, mg/dl, mean (SD)	193.6 (38.1)	192.8 (36.1)	190.7 (36.7)	194.2 (38.4)	196.7 (40.9)	<0.001
TG, mg/dl, mean (SD)	131.3 (94.3)	87.6 (41.5)	107.2 (57.6)	132.2 (71.0)	198.8 (136.1)	<0.001
FBG, mg/dl, mean (SD)	110.2 (36.7)	104.0 (31.6)	107.5 (35.9)	110.4 (36.1)	118.8 (40.9)	<0.001
LDL, mg/dl, mean (SD)	116.7 (35.0)	113.4 (32.1)	116.7 (31.7)	121.0 (34.9)	115.6 (40.1)	<0.001
BUN, mg/dl, mean (SD)	15.8 (4.6)	15.7 (4.6)	15.7 (4.5)	15.6 (4.4)	16.0 (4.9)	0.006
Cr, mg/dl, mean (SD)	0.8 (0.2)	0.7 (0.2)	0.8 (0.2)	0.8 (0.3)	0.9 (0.3)	<0.001
CRP, mg/l, mean (SD)	2.7 (7.6)	2.0 (5.5)	2.8 (9.0)	2.6 (7.1)	3.6 (8.1)	
Smoking, n (%)						0.026
No	3358 (39.3%)	892(41.6%)	846(39.7%)	794(37.2%)	826(38.8%)	
Yes	5179 (60.7%)	1252(58.4%)	1283(60.3%)	1343(62.8%)	1301(61.2%)	
Alcohol Use, n (%)						<0.001
No	2796 (32.8%)	786(36.7%)	706(33.2%)	653(30.6%)	651(30.6%)	
Yes	5741 (67.2%)	1358(63.3%)	1423(66.8%)	1484(69.4%)	1476(69.4%)	
Obesity, n (%)						<0.001
No	7561 (88.6%)	2134(99.5%)	2062(96.9%)	1901(89.0%)	1464(68.8%)	
Yes	976 (11.4%)	10(0.5%)	67(3.1%)	236(11.0%)	663(31.2%)	
Diabetes, n (%)						<0.001
No	8052 (94.3%)	2082(97.1%)	2045(96.1%)	2010(94.1%)	1915(90.0%)	
Yes	485 (5.7%)	62(2.9%)	84(3.9%)	127(5.9%)	212(10.0%)	
Kidney Diseases, n (%)						0.373
No	7972 (93.4%)	1986(92.6%)	1988(93.4%)	2007(93.9%)	1991(93.6%)	
Yes	565 (6.6%)	158(7.4%)	141(6.6%)	130(6.1%)	136(6.4%)	
Cancers, n (%)						0.433
No	8461 (99.1%)	2129(99.3%)	2107(99.0%)	2121(99.3%)	2104(98.9%)	
Yes	76 (0.9%)	15(0.7%)	22(1.0%)	16(0.7%)	23(1.1%)	
Stroke, n (%)						<0.001
No	8369 (98.0%)	2122(99.0%)	2096(98.4%)	2093(97.9%)	2058(96.8%)	
Yes	168 (2.0%)	22(1.0%)	33(1.6%)	44(2.1%)	69(3.2%)	
CVD, n (%)						<0.001
No	7535 (88.3%)	1958(91.3%)	1902(89.3%)	1884(88.2%)	1791(84.2%)	
Yes	1002 (11.7%)	186(8.7%)	227(10.7%)	253(11.8%)	336(15.8%)	
Sarcopenia, n (%)						<0.001
No	7455 (87.3%)	1995(93.1%)	1898(89.1%)	1844(86.3%)	1718(80.8%)	
Yes	1082 (12.7%)	149(6.9%)	231(10.9%)	293(13.7%)	409(19.2%)	

UHR, uric acid to high-density lipoprotein cholesterol ratio; BRI, body roundness index; TG, triglyceride; FBG, fasting blood glucose; LDL, low density lipoprotein; BUN, blood urea nitrogen; Cr, creatinine; CRP, C-reactive protein; CVD, cardiovascular disease; SD, standard deviation; CHARLS, China Health and Retirement Longitudinal Study.

**Table 2.** Association between UHR-BRI and the risk of sarcopenia.

Cohort	Exposure	Model1		Model2		Model3	
		OR (95%CI)	P-value	OR (95%CI)	P-value	OR (95%CI)	P-value
CHARLS							
	Per 1-SD increment	3.36 (2.74–4.10)	<0.001	3.29 (2.65–4.08)	<0.001	3.29 (2.64–4.10)	<0.001
	Groups						
	cuUHR-BRI Q1	Ref		Ref		Ref	
	cuUHR-BRI Q2	1.63 (1.32–2.02)	<0.001	1.70 (1.36–2.13)	<0.001	1.69 (1.35–2.12)	<0.001
	cuUHR-BRI Q3	2.13 (1.73–2.62)	<0.001	2.11 (1.70–2.63)	<0.001	2.09 (1.69–2.61)	<0.001
	cuUHR-BRI Q4	3.19 (2.62–3.90)	<0.001	3.04 (2.47–3.75)	<0.001	3.01 (2.45–3.72)	<0.001
	P for trend	<0.001		<0.001		<0.001	
NHANES							
	Per 1-SD increment	4.10(3.55–4.74)	<0.001	4.02(3.57–4.63)	<0.001	3.97(3.47–4.55)	<0.001
	Groups						
	UHR-BRI Q1	Ref		Ref		Ref	
	UHR-BRI Q2	4.30 (2.81–6.83)	<0.001	4.06 (2.65–6.46)	<0.001	3.97 (2.59–6.32)	<0.001
	UHR-BRI Q3	9.10 (6.11–14.18)	<0.001	8.50 (5.68–13.28)	<0.001	8.10 (5.41–12.68)	<0.001
	UHR-BRI Q4	18.42 (12.5–28.45)	<0.001	17.91 (12.1–27.77)	<0.001	17.14 (11.55–26.60)	<0.001
	P for trend	<0.001		<0.001		<0.001	

Model 1: No adjustment.

Model 2: Adjusted for age, sex.

Model 3: Further adjusted for marital status, educational level, TG, FBG, Cr, BUN, smoking and alcohol use.

UHR, uric acid to high-density lipoprotein cholesterol ratio; BRI, body roundness index; OR, odds ratio; CI, confidence interval; CHARLS, China Health and Retirement Longitudinal Study; NHANES, National Health and Nutrition Examination Survey.

**Table 4.** Association between UHR-BRI and the risk of sarcopenia comorbidity in the NHANES cohort.

Outcome	Exposure	Model1		Model2		Model3	
		OR (95%CI)	P-value	OR (95%CI)	P-value	OR (95%CI)	P-value
Sarcopenic obesity							
	Per 1-SD increment	6.48 (5.49–7.67)	<0.001	6.43 (5.47–7.58)	<0.001	5.86 (5.02–6.85)	<0.001
	Groups						
	UHR-BRI Q1	Ref		Ref		Ref	
	UHR-BRI Q2	8.90 (5.78–16.33)	<0.001	8.47 (5.71–15.15)	<0.001	8.01 (5.62–13.41)	<0.001
	UHR-BRI Q3	11.52 (9.54–13.02)	0.002	9.16 (8.07–12.20)	<0.001	10.23 (8.08–11.57)	<0.001
	UHR-BRI Q4	24.12 (14.3–27.62)	<0.001	21.05 (12.61–23.14)	0.035	17.03 (10.61–20.99)	<0.001
	P for trend	0.001		<0.001		<0.001	
S-KD comorbidity							
	Per 1-SD increment	2.29 (1.33–3.5)	<0.001	2.16 (1.24–3.35)	0.001	1.67 (0.81–3.43)	0.156
	Groups						
	UHR-BRI Q1	Ref		Ref		Ref	
	UHR-BRI Q2	2.02 (1.81–2.68)	<0.001	1.82 (1.42–1.96)	0.002	1.50 (1.06–1.78)	0.006
	UHR-BRI Q3	6.14 (4.36–8.87)	<0.001	5.78 (3.64–6.88)	<0.001	4.92 (2.96–6.09)	0.002
	UHR-BRI Q4	8.78 (5.06–10.92)	<0.001	6.84 (5.67–9.18)	<0.001	5.61 (4.11–7.97)	<0.001
	P for trend	0.001		<0.001		<0.001	
S-DM comorbidity							
	Per 1-SD increment	3.41 (2.60–4.44)	<0.001	3.32 (2.55–4.31)	<0.001	3.27 (2.57–4.14)	<0.001
	Groups						
	UHR-BRI Q1	Ref		Ref		Ref	
	UHR-BRI Q2	3.28 (1.16–6.66)	0.038	2.57 (0.90–5.15)	0.101	2.49 (0.91–4.23)	0.095
	UHR-BRI Q3	6.08 (2.34–8.72)	<0.001	4.54 (1.74–6.54)	0.005	3.55 (1.24–5.60)	0.005
	UHR-BRI Q4	18.93 (7.84–24.23)	<0.001	15.38 (6.30–20.88)	<0.001	12.6 (5.36–21.72)	<0.001
	P for trend	0.001		<0.001		<0.001	
S-CVD comorbidity							
	Per 1-SD increment	3.20 (2.34–4.33)	<0.001	3.08 (2.18–4.29)	<0.001	2.97 (2.09–4.18)	<0.001
	Groups						
	UHR-BRI Q1	Ref		Ref		Ref	
	UHR-BRI Q2	2.28 (1.26–3.81)	0.002	2.07 (1.11–4.15)	0.009	1.68 (0.90–3.23)	0.12
	UHR-BRI Q3	5.10 (3.95–6.81)	<0.001	4.01 (2.74–5.60)	0.024	2.18 (1.82–4.59)	0.002
	UHR-BRI Q4	8.03 (5.16–11.20)	<0.001	5.18 (3.09–8.62)	<0.001	5.19 (3.36–7.91)	<0.001
	P for trend	<0.001		<0.001		<0.001	
Sarcopenia-Stroke comorbidity							
	Per 1-SD increment	2.72 (1.63–4.31)	<0.001	2.72 (1.65–4.16)	<0.001	2.58 (1.56–4.06)	<0.001
	Groups						
	UHR-BRI Q1	Ref		Ref		Ref	
	UHR-BRI Q2	4.13 (2.90–8.18)	<0.001	3.87 (3.11–5.23)	0.001	2.10 (1.82–4.13)	0.003
	UHR-BRI Q3	8.02 (6.12–10.08)	<0.001	6.98 (5.74–9.60)	0.002	5.09 (3.95–7.31)	<0.001
	UHR-BRI Q4	11.03 (7.99–14.20)	<0.001	9.92 (6.09–11.62)	<0.001	8.19 (5.36–9.91)	<0.001
	P for trend	<0.001		<0.001		<0.001	
Sarcopenia-Cancer comorbidity							
	Per 1-SD increment	2.86 (1.94–4.16)	<0.001	2.70 (1.85–3.91)	<0.001	2.66 (1.80–3.86)	<0.001
	Groups						
	UHR-BRI Q1	Ref		Ref		Ref	
	UHR-BRI Q2	7.05 (5.25–11.90)	0.002	6.04 (4.07–11.07)	0.013	5.69 (3.55–10.72)	0.024
	UHR-BRI Q3	12.11 (8.65–20.69)	<0.001	10.95 (7.14–22.08)	<0.001	10.38 (6.01–19.13)	<0.001
	UHR-BRI Q4	19.23 (12.11–25.77)	0.004	15.34 (10.02–20.31)	<0.001	14.29 (9.5–17.15)	<0.001
	P for trend	<0.001		<0.001		<0.001	

Model 1: No adjustment.

Model 2: Adjusted for age, sex.

Model 3: Further adjusted for marital status, educational level, TG, FBG, Cr, BUN, smoking and alcohol use.

UHR, uric acid to high-density lipoprotein cholesterol ratio; BRI, body roundness index; S-KD, sarcopenia-kidney disease; S-DM, sarcopenia-diabetes mellitus, S-CVD, sarcopenia-cardiovascular disease; OR, odds ratio; CI, confidence interval; NHANES, National Health and Nutrition Examination Survey.

**Table 5.** Association between OBS and the risk of sarcopenia and sarcopenia comorbidity.

Outcome	Exposure	Model1		Model2		Model3	
		OR (95%CI)	P-value	OR (95%CI)	P-value	OR (95%CI)	P-value
Sarcopenia							
	Per 1-SD increment	0.70 (0.66–0.74)	<0.001	0.70 (0.66–0.75)	<0.001	0.69 (0.64–0.73)	<0.001
	Groups						
	UHR-BRI Q1	Ref		Ref		Ref	
	UHR-BRI Q2	0.60 (0.50–0.72)	<0.001	0.60 (0.50–0.72)	<0.001	0.57 (0.47–0.69)	<0.001
	UHR-BRI Q3	0.37 (0.30–0.46)	<0.001	0.38 (0.30–0.47)	<0.001	0.36 (0.29–0.45)	<0.001
	UHR-BRI Q4	0.22 (0.16–0.30)	<0.001	0.23 (0.17–0.31)	<0.001	0.21 (0.15–0.28)	<0.001
	P for trend	<0.001		<0.001		<0.001	
Sarcopenic obesity							
	Per 1-SD increment	0.53 (0.49–0.58)	<0.001	0.54 (0.50–0.59)	<0.001	0.63 (0.59–0.67)	<0.001
	Groups						
	UHR-BRI Q1	Ref		Ref		Ref	
	UHR-BRI Q2	0.46 (0.37–0.56)	0.004	0.46 (0.37–0.56)	<0.001	0.48 (0.40–0.60)	<0.001
	UHR-BRI Q3	0.12 (0.08–0.17)	<0.001	0.14 (0.08–0.19)	0.017	0.17 (0.10–0.24)	<0.001
	UHR-BRI Q4	0.06 (0.03–0.10)	0.021	0.08 (0.04–0.11)	<0.001	0.08 (0.05–0.13)	<0.001
	P for trend	0.001		<0.001		0.023	
S-KD comorbidity							
	Per 1-SD increment	0.79 (0.58–0.83)	0.022	0.82 (0.61–0.89)	<0.001	0.89 (0.69–1.10)	0.068
	Groups						
	UHR-BRI Q1	Ref		Ref		Ref	
	UHR-BRI Q2	0.77 (0.30–1.21)	0.068	0.78 (0.3–1.62)	0.083	0.98 (0.47–1.98)	0.062
	UHR-BRI Q3	0.43 (0.17–0.83)	0.023	0.67 (0.18–0.98)	0.041	0.78 (0.39–1.12)	0.083
	UHR-BRI Q4	0.11 (0.01–0.56)	<0.001	0.12 (0.01–0.60)	<0.001	0.24 (0.20–0.73)	0.005
	P for trend	<0.001		0.003		0.001	
S-DM comorbidity							
	Per 1-SD increment	0.60(0.51–0.70)	<0.001	0.61 (0.52–0.71)	<0.001	0.66 (0.53–0.77)	<0.001
	Groups						
	UHR-BRI Q1	Ref		Ref		Ref	
	UHR-BRI Q2	0.53 (0.34–0.81)	0.004	0.57 (0.34–0.87)	0.005	0.63 (0.44–0.99)	0.003
	UHR-BRI Q3	0.23 (0.12–0.40)	<0.001	0.27 (0.12–0.44)	<0.001	0.34 (0.12–0.54)	<0.001
	UHR-BRI Q4	0.19 (0.08–0.38)	<0.001	0.21 (0.09–0.43)	<0.001	0.27 (0.09–0.49)	<0.001
	P for trend	<0.001		0.003		0.001	
S-CVD comorbidity							
	Per 1-SD increment	0.56 (0.45–0.69)	<0.001	0.56 (0.45–0.70)	<0.001	0.58 (0.46–0.72)	<0.001
	Groups						
	UHR-BRI Q1	Ref		Ref		Ref	
	UHR-BRI Q2	0.51 (0.27–0.92)	0.028	0.51 (0.27–0.92)	0.031	0.53 (0.28–0.96)	0.04
	UHR-BRI Q3	0.11 (0.03–0.30)	<0.001	0.12 (0.03–0.33)	<0.001	0.12 (0.03–0.35)	<0.001
	UHR-BRI Q4	0.15 (0.04–0.43)	0.002	0.17 (0.04–0.47)	0.003	0.19 (0.04–0.53)	0.006
	P for trend	<0.001		0.003		0.001	
Sarcopenia-Stroke comorbidity							
	Per 1-SD increment	0.51 (0.35–0.73)	<0.001	0.52 (0.35–0.74)	<0.001	0.53 (0.37–0.77)	<0.001
	Groups						
	UHR-BRI Q1	Ref		Ref		Ref	
	UHR-BRI Q2	0.61 (0.23–1.54)	0.31	0.62 (0.23–1.54)	0.311	0.64 (0.24–1.59)	0.344
	UHR-BRI Q3	0.49 (0.26–0.92)	0.017	0.52 (0.34–0.79)	0.026	0.59 (0.39–0.94)	0.048
	UHR-BRI Q4	0.14 (0.01–0.73)	0.042	0.18 (0.01–0.9)	0.006	0.24 (0.01–0.81)	0.033
	P for trend	0.003		0.004		0.003	
Sarcopenia-Cancer comorbidity							
	Per 1-SD increment	0.67 (0.52–0.85)	0.002	0.71 (0.55–0.91)	0.008	0.72 (0.56–0.93)	0.014
	Groups						
	UHR-BRI Q1	Ref		Ref		Ref	
	UHR-BRI Q2	0.55 (0.26–1.13)	0.113	0.58 (0.27–1.18)	0.141	0.59 (0.27–1.22)	0.164
	UHR-BRI Q3	0.23 (0.07–0.6)	0.007	0.33 (0.08–0.97)	0.013	0.34 (0.08–1.04)	0.092
	UHR-BRI Q4	0.24 (0.06–0.71)	0.023	0.26 (0.07–0.68)	0.043	0.28 (0.08–0.74)	0.028
	P for trend	0.001		0.005		0.009	

Model 1: No adjustment.

Model 2: Adjusted for age, sex.

Model 3: Further adjusted for marital status, educational level, TG, FBG, Cr, BUN, smoking and alcohol use.

UHR, uric acid to high-density lipoprotein cholesterol ratio; BRI, body roundness index; OBS, oxidative balance score; S-KD, sarcopenia-kidney disease; S-DM, sarcopenia- diabetes mellitus, S-CVD, sarcopenia-cardiovascular disease; OR, odds ratio; CI, confidence interval.

### The combined effect of UHR and BRI on sarcopenia

Logistic regression analyses revealed that, relative to the Type 1 reference group, participants in Type 8 had a 10.30-fold higher risk of sarcopenia in the CHARLS cohort (OR: 10.30, 95% CI: 7.28–14.59). In the NHANES cohort, participants in Type 8 had a 30.38-fold higher risk of sarcopenia compared with those in Type 1 (OR: 30.38, 95% CI: 17.02–56.34). The p-values for trend across subgroups were statistically significant in both cohorts, indicating a robust association of UHR and BRI with the risk of sarcopenia (Table S6, S7).

### Interaction between UHR and BRI on sarcopenia

Interaction analysis revealed a significant multiplicative interaction between UHR and BRI. In the CHARLS cohort, the multiplicative interaction estimate for sarcopenia was 1.25 (95% CI: 1.20–1.39). In the NHANES cohort, the corresponding value was 1.20 (95% CI: 1.04–1.37) for sarcopenia, indicating a synergistic risk amplification effect. However, the 95% confidence intervals for RERI, AP, and SI all crossed the null value of 0 or 1, suggesting that the additive interaction was not statistically significant (Table S8, S9). Based on these findings, we constructed a multiple composite risk index, UHR-BRI, by multiplying UHR and BRI for subsequent analyses.

### Baseline characteristics of participants stratified by UHR-BRI quartiles

Participants were categorized into groups according to the quartiles of UHR-BRI, and the baseline characteristics of the participants are presented in Table 1 and S3. Significant differences in baseline characteristics were observed across the quartile groups, including demographic factors such as age and sex, laboratory indicators such as TG and FBG, as well as medical history of comorbidities including diabetes and cardiovascular disease. Notably, the diagnosis of sarcopenia in the highest quartile group (Q4) was significantly higher than that in the lowest quartile group (Q1) (CHARLS: 19.2% vs. 6.9%; NHANES: 17.9% vs. 1.2%).

**Table 3.** Association between UHR-BRI and the risk of sarcopenia comorbidity in the CHARLS cohort.

Outcome	Exposure	Model1		Model2		Model3	
		OR (95%CI)	P-value	OR (95%CI)	P-value	OR (95%CI)	P-value
Sarcopenic obesity							
	Per 1-SD increment	14.37 (10.37–19.96)	<0.001	13.97 (10.06–19.45)	<0.001	12.44 (10.28–19.38)	<0.001
	Groups						
	cuUHR-BRI Q1	Ref		Ref		Ref	
	cuUHR-BRI Q2	3.29 (1.16–11.68)	0.037	3.26 (1.15–11.6)	0.039	3.23 (1.14–11.5)	0.040
	cuUHR-BRI Q3	8.65 (3.44–19.02)	<0.001	8.62 (3.43–18.93)	<0.001	8.55 (3.4–18.68)	<0.001
	cuUHR-BRI Q4	17.69 (9.88–22.8)	<0.001	16.33 (8.3–18.38)	<0.001	15.83 (8.06–16.91)	<0.001
	P for trend	<0.001		<0.001		<0.001	
S-KD comorbidity							
	Per 1-SD increment	3.81 (2.06–6.52)	<0.001	3.48 (1.86–6.02)	0.001	3.18 (2.63–14.72)	0.156
	Groups						
	cuUHR-BRI Q1	Ref		Ref		Ref	
	cuUHR-BRI Q2	1.01 (0.41–2.46)	0.987	1.00 (0.41–2.44)	0.994	0.94 (0.36–2.44)	0.901
	cuUHR-BRI Q3	1.41 (0.63–3.27)	0.411	1.40 (0.62–3.26)	0.419	1.73 (0.72–4.26)	0.223
	cuUHR-BRI Q4	3.36 (1.72–7.21)	<0.001	3.13 (1.59–6.71)	0.002	4.31 (2.01–9.99)	<0.001
	P for trend	<0.001		<0.001		<0.001	
S-DM comorbidity							
	Per 1-SD increment	7.10 (4.24–11.5)	<0.001	6.90 (4.05–11.38)	<0.001	6.01 (3.48–10.06)	<0.001
	Groups						
	cuUHR-BRI Q1	Ref		Ref		Ref	
	cuUHR-BRI Q2	1.21 (0.36–4.2)	0.754	1.21 (0.36–4.19)	0.757	1.10 (0.33–3.85)	0.876
	cuUHR-BRI Q3	2.62 (0.99–8.17)	0.068	2.55 (0.96–7.95)	0.077	2.40 (0.89–7.52)	0.100
	cuUHR-BRI Q4	7.78 (3.35–22.64)	<0.001	7.12 (3.06–20.72)	<0.001	6.14 (2.62–17.96)	<0.001
	P for trend	<0.001		<0.001		<0.001	
S-CVD comorbidity							
	Per 1-SD increment	3.72 (2.38–5.61)	<0.001	3.46 (2.16–5.36)	<0.001	3.44 (2.11–5.41)	<0.001
	Groups						
	cuUHR-BRI Q1	Ref		Ref		Ref	
	cuUHR-BRI Q2	1.16 (0.63–2.14)	0.629	1.17 (0.64–2.17)	0.606	1.15 (0.63–2.13)	0.654
	cuUHR-BRI Q3	1.56 (0.89–2.79)	0.121	1.47 (0.84–2.64)	0.182	1.48 (0.84–2.67)	0.177
	cuUHR-BRI Q4	2.92 (1.78–5.00)	<0.001	2.58 (1.57–4.43)	<0.001	2.52 (1.52–4.36)	<0.001
	P for trend	<0.001		<0.001		<0.001	
Sarcopenia-Stroke comorbidity							
	Per 1-SD increment	5.14 (2.24–10.23)	<0.001	5.04 (2.07–10.66)	<0.001	5.02 (2.03–10.88)	<0.001
	Groups						
	cuUHR-BRI Q1	Ref		Ref		Ref	
	cuUHR-BRI Q2	1.34 (0.30–6.83)	0.699	1.37 (0.30–6.99)	0.679	1.39 (0.31–7.08)	0.668
	cuUHR-BRI Q3	2.35 (0.65–10.89)	0.217	2.16 (0.60–10.03)	0.267	2.19 (0.61–10.23)	0.257
	cuUHR-BRI Q4	5.07 (1.67–21.91)	0.010	4.34 (1.42–18.80)	0.021	4.32 (1.40–18.86)	0.022
	P for trend	0.002		0.006		0.007	
Sarcopenia-Cancer comorbidity							
	Per 1-SD increment	6.26 (3.75–10.06)	<0.001	6.10 (3.59–9.97)	<0.001	5.36 (3.11–8.9)	<0.001
	Groups						
	cuUHR-BRI Q1	Ref		Ref		Ref	
	cuUHR-BRI Q2	1.01 (0.34–2.95)	0.990	1.00 (0.34–2.94)	0.993	0.90 (0.31–2.66)	0.850
	cuUHR-BRI Q3	2.16 (0.91–5.66)	0.094	2.09 (0.88–5.50)	0.108	1.96 (0.82–5.17)	0.146
	cuUHR-BRI Q4	5.70 (2.71–13.96)	<0.001	5.24 (2.49–12.84)	<0.001	4.48 (2.11–11.05)	<0.001
	P for trend	<0.001		<0.001		<0.001	

Model 1: No adjustment.

Model 2: Adjusted for age, sex.

Model 3: Further adjusted for marital status, educational level, TG, FBG, Cr, BUN, smoking and alcohol use.

UHR, uric acid to high-density lipoprotein cholesterol ratio; BRI, body roundness index; S-KD, sarcopenia-kidney disease; S-DM, sarcopenia-diabetes mellitus, S-CVD, sarcopenia-cardiovascular disease; OR, odds ratio; CI, confidence interval; CHARLS, China Health and Retirement Longitudinal Study.

### Evaluation of UHR-BRI for sarcopenia

**Figure 1.**
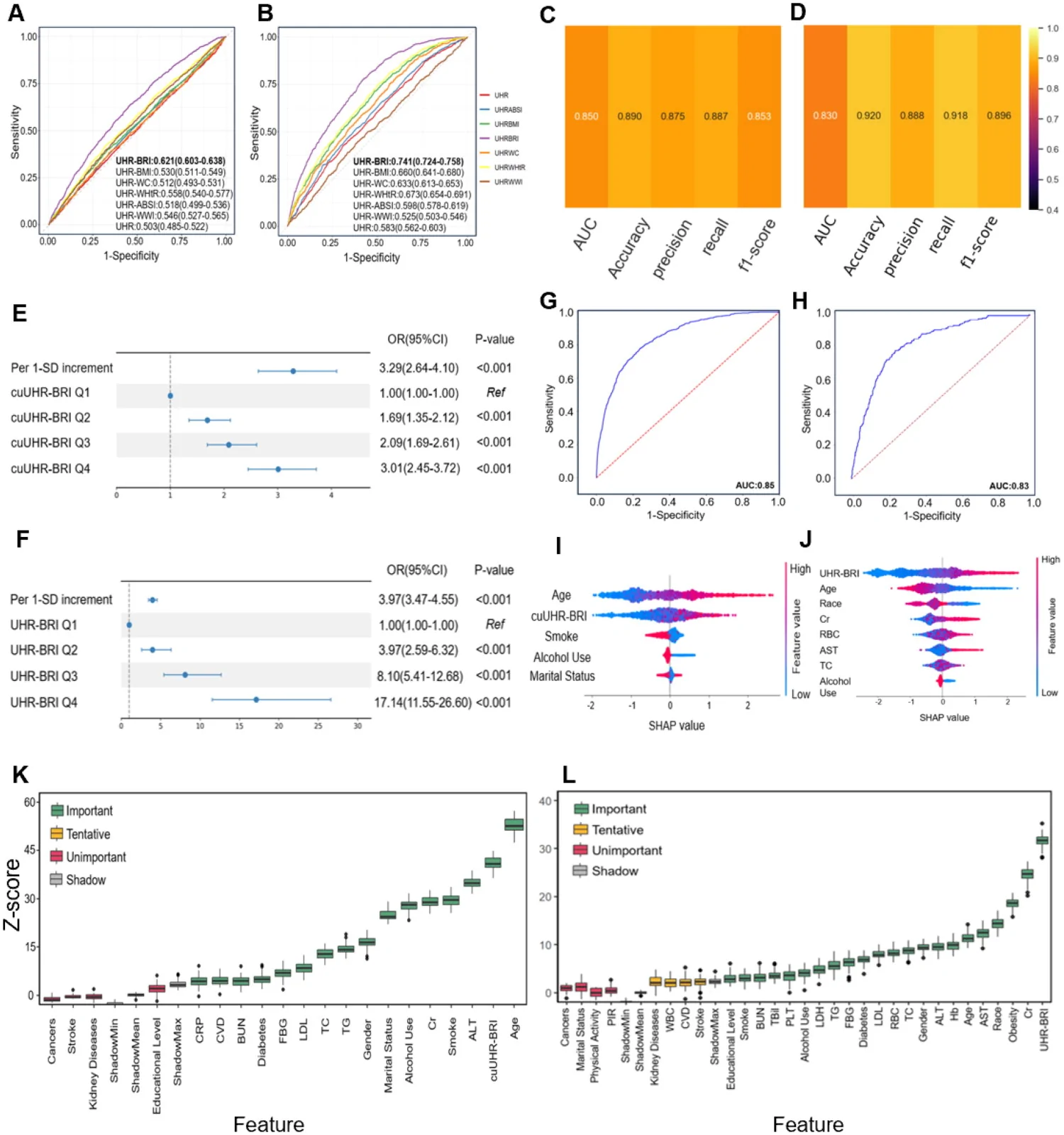
Association analysis of UHR-BRI for sarcopenia. (A) ROC curves of UHR-BRI and other indices for sarcopenia in the CHARLS cohort, (B) ROC curves of UHR-BRI and other indices for sarcopenia in the NHANES cohort, (C) Performance metrics of the XGBoost model in the CHARLS cohort, (D) Performance metrics of the XGBoost model in the NHANES cohort, (E) Association between UHR-BRI and sarcopenia in the CHARLS cohort: logistic regression forest plot (Model3), (F) Association between UHR-BRI and sarcopenia in the NHANES cohort: logistic regression forest plot (Model3), (G) ROC curve of the XGBoost model for sarcopenia risk prediction in the CHARLS cohort, (H) ROC curve of the XGBoost model for sarcopenia risk prediction in the NHANES cohort, (I) SHAP values analysis interpreting feature contributions in the CHARLS cohort, (J) SHAP values analysis interpreting feature contributions in the NHANES cohort, (K)Boruta algorithm-based feature selection for identifying key predictors of sarcopenia in the CHARLS cohort, (L)Boruta algorithm-based feature selection for identifying key predictors of sarcopenia in the NHANES cohort. UHR, uric acid to high-density lipoprotein cholesterol ratio; BRI, body roundness index; BMI, body mass index; WC, waist circumference; WHtR, waist-to-height ratio; ABSI, a body shape index; WWI, waist-to-weight index; PIR, poverty income ratio; TG, triglyceride; FBG, fasting blood glucose; LDL, low density lipoprotein; WBC, white blood cell; RBC, red blood cell; Hb, hemoglobin; PLT, platelet; ALT, alanine aminotransferase; AST, aspartate aminotransferase; BUN, blood urea nitrogen; LDH, lactate dehydrogenase; TBil, total bilirubin; TC, total cholesterol; Cr, creatinine; CRP, C-reactive protein; CVD, cardiovascular disease; ROC, receiver operating characteristic; OR, odds ratio; SD, standard deviation; CI, confidence interval; NHANES, National Health and Nutrition Examination Survey; CHARLS, China Health and Retirement Longitudinal Study.

**Figure 2.**
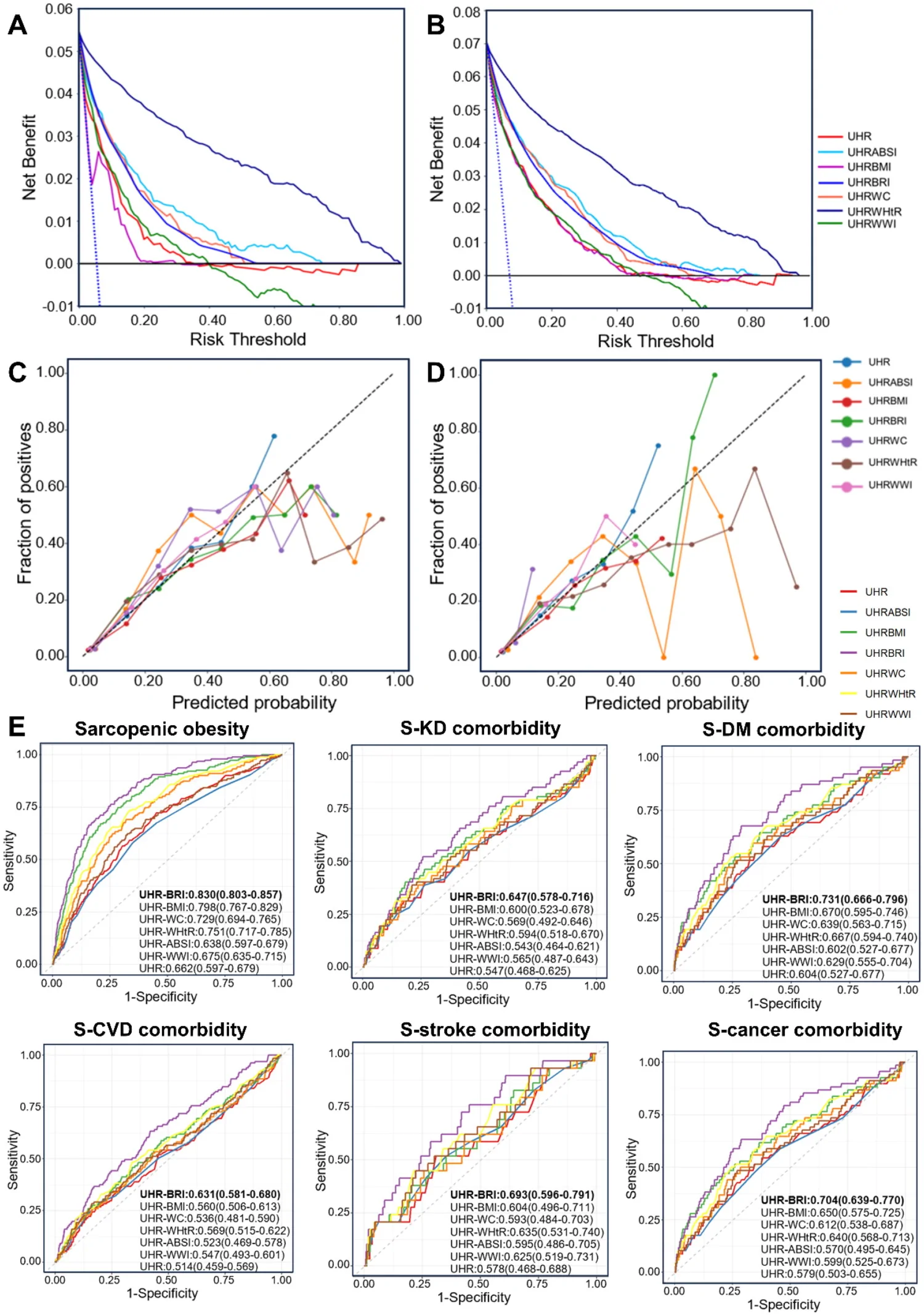
Diagnostic performance of UHR-BRI for sarcopenia. (A) DCA of UHR-BRI and other indices for sarcopenia in the CHARLS cohort, (B) DCA of UHR-BRI and other indices for sarcopenia in the NHANES cohort, (C) Calibration curves of UHR-BRI and other indices for sarcopenia in the CHARLS cohort, (D) Calibration curves of UHR-BRI and other indices for sarcopenia in the NHANES cohort, (E) ROC curves of UHR-BRI and other indices for sarcopenia comorbidities in the CHARLS cohort. UHR, uric acid to high-density lipoprotein cholesterol ratio; BRI, body roundness index; BMI, body mass index; WC, waist circumference; WHtR, waist-to-height ratio; ABSI, a body shape index; WWI, waist-to-weight index; DCA, decision curve analysis; ROC, receiver operating characteristic; S-KD, sarcopenia-kidney disease; S-DM, sarcopenia-diabetes mellitus, S-CVD, sarcopenia-cardiovascular disease; S-stroke, sarcopenia-stroke; S-cancer, sarcopenia-cancer; NHANES, National Health and Nutrition Examination Survey; CHARLS, China Health and Retirement Longitudinal Study.

ROC analysis demonstrated that UHR-BRI exhibited superior AUC values relative to other indicators across the two cohorts (CHARLS AUC: 0.621, 95% CI: 0.603–0.638; NHANES AUC: 0.741, 95% CI: 0.724–0.758) (Figure 1A, 1B, S2). Notably, the ROC analysis merely assessed the correlation between single indicators and sarcopenia and did not incorporate additional clinical risk factors, accordingly, the resulting AUC values are relatively modest. Furthermore, compared with the baseline model, the addition of UHR-BRI resulted in a significant improvement in the net reclassification improvement (NRI) and an enhancement in the integrated discrimination improvement (IDI) (Table S10, S11). In addition, decision curve analysis and calibration curves further supported this conclusion (Figure 2A-D).

### Association between UHR‑BRI exposure trajectories and sarcopenia

**Figure 3.**
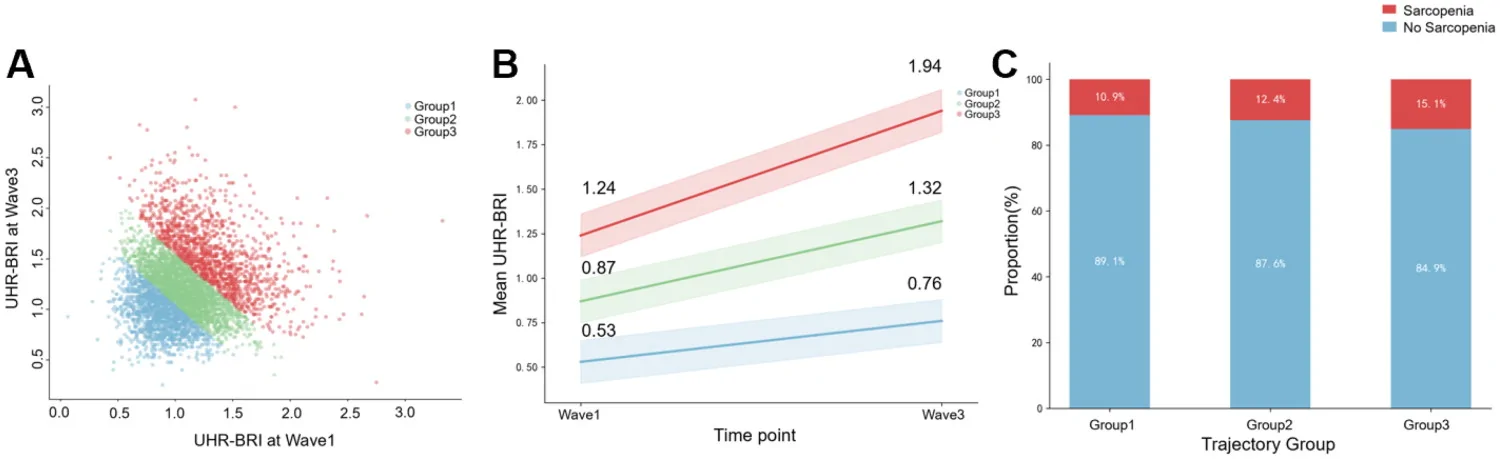
GBTM analysis of UHR-BRI across study waves. (A) Scatter plots of individual observed UHR-BRI values across study waves, (B) Fitted longitudinal trajectory curves of UHR-BRI, (C)Bar charts displaying the sarcopenia stratified by trajectory group of UHR-BRI. GBTM, group-based trajectory modeling; UHR, uric acid to high-density lipoprotein cholesterol ratio; BRI, body roundness index.

After identifying the optimal performance of the UHR‑BRI index, trajectory analysis of UHR‑BRI exposure in the CHARLS cohort identified three distinct trajectory patterns: Group 1 (low‑stable), Group 2 (moderate‑stable), and Group 3 (high‑stable). The prevalence of sarcopenia showed an increasing trend across groups, at 10.9%, 12.4%, and 15.1%, respectively. Overall, the group with the highest trajectory was associated with the greatest risk of incident sarcopenia (Figure 3). Accordingly, we calculated cumulative UHR‑BRI for subsequent analyses. Analyses of exposure trajectories for UHR and BRI in relation to sarcopenia are also presented in Figure S5.

### Association between UHR-BRI and the risk of sarcopenia

We analyzed the association between UHR-BRI and sarcopenia using the previously constructed logistic regression models. In the CHARLS cohort, after adjusting for potential confounders, each one-standard-deviation increase in cuUHR-BRI was associated with a 3.29-fold higher risk of sarcopenia (OR = 3.29, 95% CI: 2.64–4.10). When participants were categorized according to cuUHR-BRI quartiles, compared with those in the Q1, participants in Q2, Q3, and Q4 had significantly elevated risks of sarcopenia, with odds ratios of 1.69 (1.35–2.12), 2.09 (1.69–2.61), and 3.01 (2.45–3.72), respectively, and all differences were statistically significant. In the NHANES cohort, each one-standard-deviation increase in UHR-BRI was associated with a 3.97-fold higher risk of sarcopenia (OR = 3.97, 95% CI: 3.47–4.55). When participants were stratified by UHR-BRI quartiles, those in the Q4 had a significantly increased risk (OR = 17.14, 95% CI: 11.55–26.60). The p-values for trend across quartiles were statistically significant in both cohorts (p for trend < 0.05), indicating a robust association between UHR-BRI levels and the risk of sarcopenia (Table 2, Figure 1E, 1F).

### Machine learning models

We subsequently constructed machine learning models, not primarily to develop prediction models, but rather to evaluate the relative importance of UHR-BRI in identifying sarcopenia among a comprehensive set of clinical and laboratory variables. We identified independent risk factors for incident sarcopenia in each of the two cohorts. In the CHARLS cohort, five variables were included, age, marital status, cuUHR-BRI, smoking, and alcohol use, in the NHANES cohort, eight variables were included, age, race/ethnicity, UHR-BRI, Cr, RBC, AST, TC, and alcohol use (Table S12, S13, Figure 1K, 1L, S6). After confirming the absence of multicollinearity through collinearity diagnostics, we constructed an XGBoost machine learning model. The predictive performance of the model is presented as a heatmap in Figure 1C and 1D (CHARLS: AUC = 0.850, accuracy = 0.890, precision = 0.875, recall = 0.887, F1 score = 0.853; NHANES: AUC = 0.830, accuracy = 0.920, precision = 0.888, recall = 0.918, F1 score = 0.896). The ROC curves, calibration curves, and decision curve analysis all confirmed the good predictive performance of the model. Subsequently, SHAP analysis was used to quantify the feature importance in the XGBoost model, and the results showed that UHR-BRI exhibited a high SHAP value, indicating its substantial contribution to the clinical outcomes (Figure 1G-J, S7-12). Collectively, these findings support UHR-BRI as a clinically relevant marker comparable to conventional clinical and laboratory indicators.

### Analyses of sarcopenia comorbidities

This study further analyzed the associations between UHR-BRI and sarcopenia accompanied by comorbidities including obesity, kidney disease, diabetes, cardiovascular disease, stroke, and cancer. First, ROC curve analysis was used to evaluate the AUC value of UHR-BRI for these comorbid outcomes, and the results showed that UHR-BRI demonstrated consistently favorable performance across these comorbid outcomes compared with the other indices (Figure 2E, S3, S4). Furthermore, using the previously constructed logistic regression models, we analyzed the association between UHR-BRI and different disease outcomes. The results indicated that as UHR-BRI levels increased, the risk of each comorbidity also increased, suggesting a significant association between UHR-BRI and sarcopenia comorbidities (Table 3, 4).

**Figure 4.**
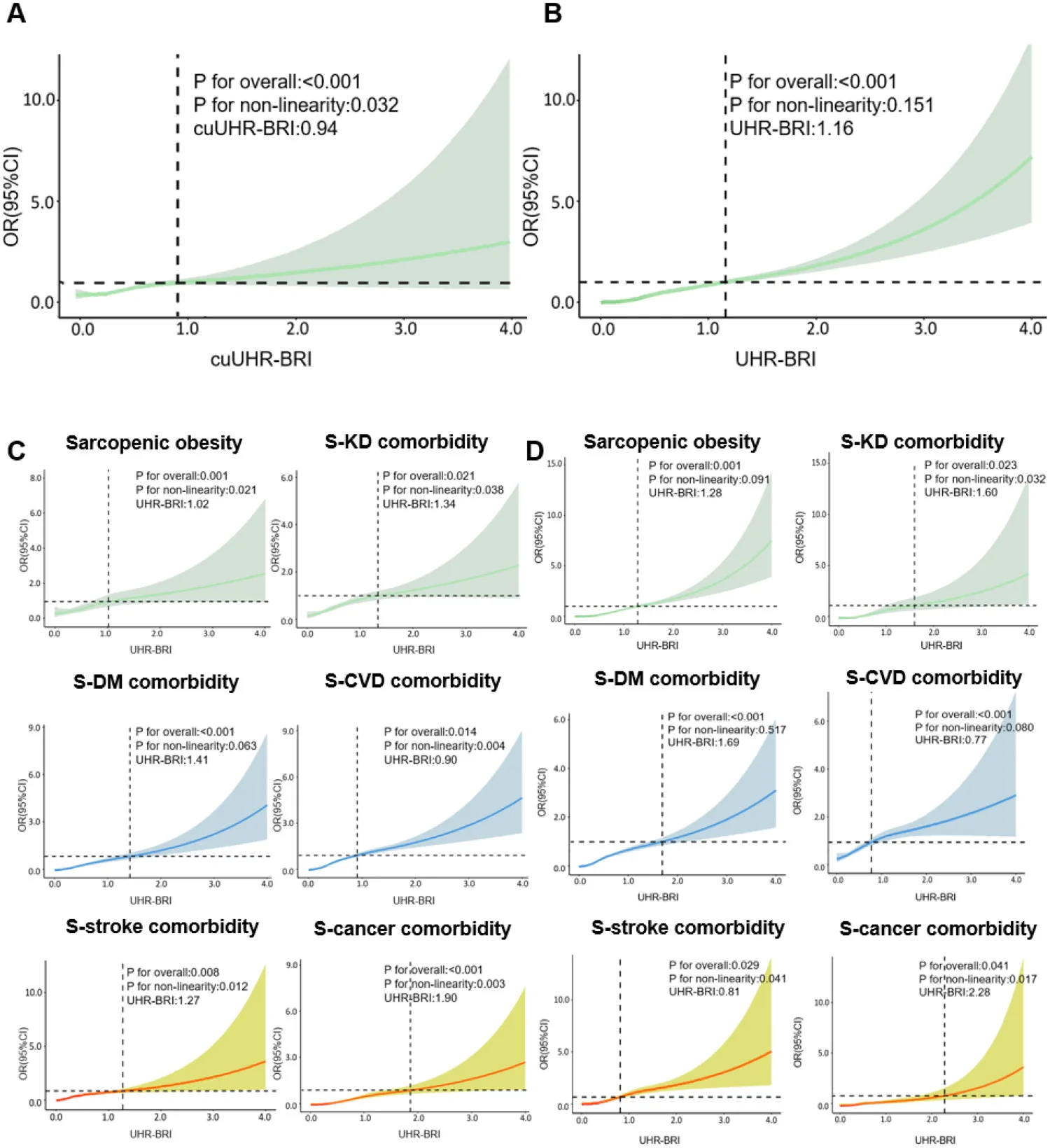
RCS Curves of UHR‑BRI with sarcopenia and sarcopenia comorbidities. (A) Dose-response association between UHR-BRI and sarcopenia in the CHARLS cohort, (B) Dose-response association between UHR-BRI and sarcopenia in the NHANES cohort, (C) Dose-response association between UHR-BRI and sarcopenia comorbidities in the CHARLS cohort, (D) Dose-response association between UHR-BRI and sarcopenia comorbidities in the NHANES cohort. UHR, uric acid to high-density lipoprotein cholesterol ratio; BRI, body roundness index; OR, odds ratio; CI, confidence interval; RCS, restricted cubic spline; S-KD, sarcopenia-kidney disease; S-DM, sarcopenia-diabetes mellitus, S-CVD, sarcopenia-cardiovascular disease; S-stroke, sarcopenia-stroke; S-cancer, sarcopenia-cancer; NHANES, National Health and Nutrition Examination Survey; CHARLS, China Health and Retirement Longitudinal Study.

### The Dose-response association of UHR-BRI with sarcopenia

To analyze the dose-response relationships between UHR-BRI and sarcopenia and its comorbidities, we performed RCS analyses. In the CHARLS cohort, a nonlinear dose-response relationship was observed between cuUHR-BRI levels and sarcopenia (p for nonlinearity < 0.05), whereas in the NHANES cohort, a linear dose-response relationship was identified (p for nonlinearity > 0.05). When analyzing sarcopenia comorbidities, we found statistically significant positive associations between UHR-BRI and the risk of sarcopenia comorbidities in both cohorts (Figure 4).

### Subgroup and interaction analyses

**Figure 5.**
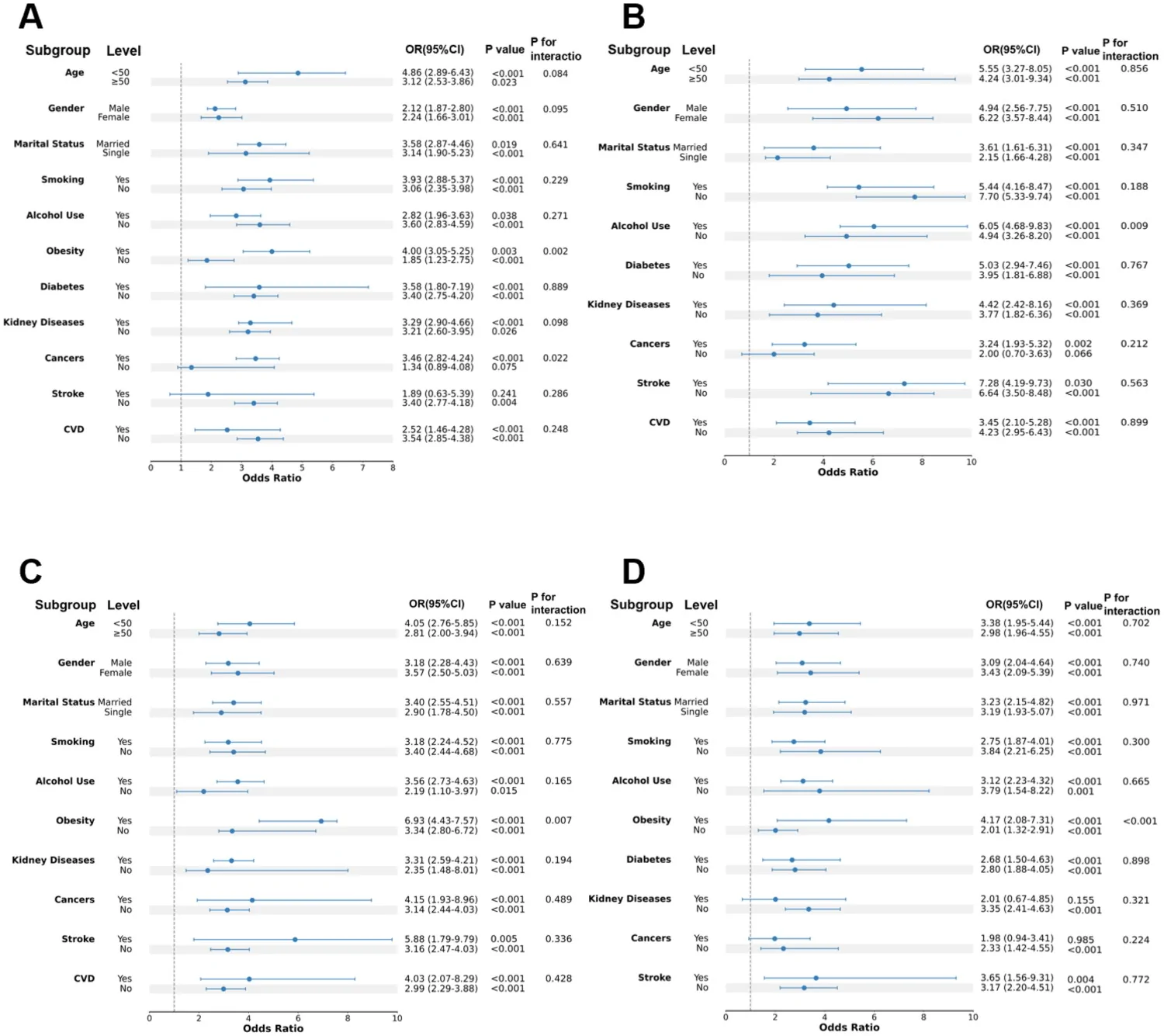
Subgroup analyses of cuUHR‑BRI with sarcopenia and sarcopenia comorbidities in the CHARLS cohort. (A) Sarcopenia, (B) Sarcopenic obesity, (C) S-DM comorbidity, (D) S-CVD comorbidity. OR, odds ratio; CI, confidence interval; S-DM, sarcopenia- diabetes mellitus, S-CVD, sarcopenia-cardiovascular disease; CHARLS, China Health and Retirement Longitudinal Study.

Subgroup analyses were performed to explore the association between UHR-BRI and sarcopenia across different population subgroups. In the CHARLS cohort, the association between cuUHR-BRI and sarcopenia was observed across most subgroups, including age, sex and so on (*p* < 0.05), with significant interactions identified in the obesity and cancer subgroups, indicating that the association was more pronounced in these populations (p for interaction < 0.05). In the NHANES cohort, the association between UHR-BRI and the risk of sarcopenia remained stable across most subgroups, but was not statistically significant in the subgroup without stroke. Furthermore, a significant interaction was observed in the cancer subgroup. In addition, we explored the associations between UHR-BRI and the comorbidities of sarcopenic obesity, diabetes, and CVD. The results demonstrated that the associations remained statistically significant across most subgroups (Figure 5, S13).

### Mediation analyses between UHR-BRI and sarcopenia

**Figure 6.**
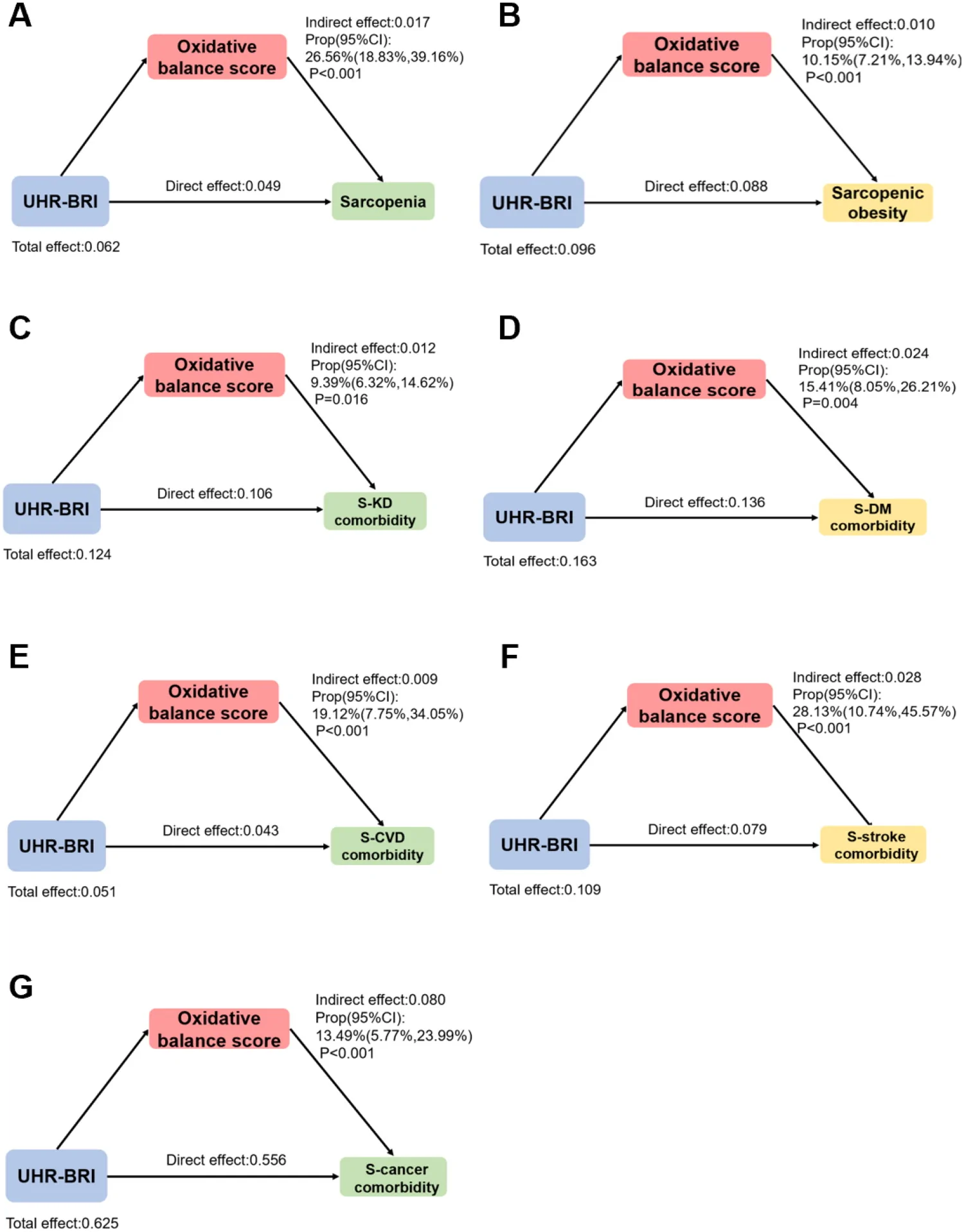
Mediation analyses the relationship between UHR-BRI, sarcopenia and sarcopenia comorbidity by OBS. (A) Sarcopenia, (B)Sarcopenic obesity, (C) S-KD comorbidity, (D) S-DM comorbidity, (E) S-CVD comorbidity, (F) S-stroke comorbidity, (G) S-cancer comorbidity. UHR, uric acid to high-density lipoprotein cholesterol ratio; BRI, body roundness index; S-KD, sarcopenia-kidney disease; S-DM, sarcopenia- diabetes mellitus, S-CVD, sarcopenia-cardiovascular disease; S-stroke, sarcopenia-stroke; S-cancer, sarcopenia-cancer; OBS, oxidative balance score; Prop, proportion.

Logistic regression results showed a significant association between OBS and sarcopenia as well as various comorbidities, indicating that the risk of each disease increased with rising OBS levels (Table 5). Mediation analysis revealed that, after full adjustment for covariates, OBS played a significant mediating role in the association between UHR-BRI and sarcopenia, with a mediation proportion of 26.56% (95% CI: 18.83%–39.16%, *p* < 0.001). Furthermore, OBS also exhibited a significant mediating effect in the association between UHR-BRI and sarcopenia comorbidities (all *p* < 0.05) (Figure 6). Notably, given the cross-sectional design of the NHANES cohort, these findings should be interpreted as exploratory evidence only and do not support causal inferences.

### Sensitivity analyses

We performed sensitivity analyses by using the complete dataset without imputation and the results showed that the association between UHR-BRI and the risk of sarcopenia remained largely consistent, indicating that the primary results were robust (Table S14, S15).

## Discussion

This study constructed a composite risk index, UHR‑BRI, which integrates purine metabolism, dyslipidemia, and the severity of central obesity, and evaluated its performance using data from two large‑scale cohorts, CHARLS and NHANES. Our findings demonstrate that: (1) higher UHR-BRI levels were significantly associated with increased risk of developing sarcopenia; (2) the associations of UHR‑BRI with sarcopenia and sarcopenia comorbidities were robust across various analyses; and (3) mediation analysis revealed that OBS mediated the association of UHR‑BRI with sarcopenia and its comorbidities.

As a composite index, the UHR is calculated as the ratio of uric acid to HDL‑C and comprehensively reflects disturbances in purine and lipid metabolism [24]. The inflammatory state reflected by high UHR can activate the NLRP3 inflammasome, promoting the release of interleukin‑1β and interleukin‑18, thereby inducing chronic low‑grade inflammation [25]. Simultaneously, it inhibits endothelial nitric oxide synthase (eNOS) activity, leading to endothelial dysfunction, which in turn compromises blood perfusion and nutrient supply to muscle tissue [26]. Such imbalance accelerates muscle protein catabolism and inhibits anabolism, serving as a key driver of sarcopenia progression [27].

The continuous rise in global obesity rates has become a major public health challenge worldwide, and obesity has been recognized to be closely associated with a variety of chronic diseases [28,29]. Central obesity, in particular, leads to excessive deposition of visceral adipose tissue, which secretes large amounts of adipokines and inflammatory mediators, thereby inducing a state of chronic inflammation and insulin resistance [30], and consequently reducing the rate of muscle protein synthesis [31]. The BRI was proposed by Thomas et al. in 2013 [32], based on body geometric analysis, this indicator focuses on the association between abdominal fat distribution and height, and has been validated as an excellent indicator for evaluating central obesity and visceral fat content [33,34]. Yang et al. based on a cross-continental cohort study involving Chinese and American populations, concluded that BRI could independently predict the risk of sarcopenia in patients with cancer, demonstrating its clinical utility [35]. Furthermore, other studies have found that BRI exhibits significant advantages in predicting sarcopenia risk in older adults [14] as well as muscle mass loss in patients with diabetes and obesity [15]. Against this background, the present study combined the UHR with BRI to construct a multiple composite risk index, UHR-BRI. The index simultaneously incorporates disturbances in purine metabolism, lipid metabolism, and obesity, enabling a more comprehensive evaluation of sarcopenia risk.

The present study demonstrated a robust positive association between UHR-BRI levels and the risk of sarcopenia. The convergence of purine metabolism disorders, dyslipidemia, and central obesity on mitochondrial dysfunction may represent a unifying mechanism underlying the strong predictive performance of UHR-BRI. The AMPK/SIRT1/PGC-1α signaling axis, which serves as a critical hub sensing cellular energy status and coordinating mitochondrial biogenesis, is profoundly affected by metabolic derangements [36,37]. Impairment of this axis leads to reduced oxidative capacity, accumulation of damaged mitochondria, and insufficient ATP production to meet the energy demands of muscle contraction and repair. The association between UHR-BRI and sarcopenia may therefore reflect the cumulative burden of mitochondrial dysfunction across multiple metabolic domains [38]. Furthermore, studies have shown that chronic inflammation can compromise the integrity of motor neurons and the neuromuscular junction (NMJ), the interface between motor nerve terminals and muscle fibers. Deterioration of NMJ function reduces neural input to muscles, leading to denervation‑induced muscle atrophy, which is an important mechanism underlying age‑related sarcopenia [39,40]. Elevated UHR-BRI may accelerate this degenerative process. However, given the observational nature of the present study, the mechanisms proposed above remain speculative and require further experimental validation.

While much attention has been given to accelerated protein degradation in sarcopenia, growing evidence indicates that impaired muscle regeneration and satellite cell dysfunction play equally important roles, particularly in the context of chronic metabolic stress [41]. The oxidative and inflammatory environment associated with elevated UHR-BRI may compromise the regenerative capacity of skeletal muscle by disrupting satellite cell quiescence, activation, and differentiation. Furthermore, aberrant mechanotransduction through Piezo1, a recently identified mechanosensitive channel that regulates myogenesis and satellite cell function, may be affected by the metabolic derangements captured by UHR-BRI [42]. This perspective suggests that interventions targeting UHR-BRI elevation might benefit muscle health not only by reducing degradation but also by preserving regenerative potential.

The clinical significance of UHR-BRI extends beyond risk stratification to potential therapeutic implications. IL-6, a pleiotropic cytokine that exhibits both physiological and pathological roles in skeletal muscle, may serve as a key effector molecule linking UHR-BRI elevation to muscle atrophy [43]. In contrast to its beneficial metabolic effects during acute exercise, chronic IL-6 elevation activates JAK/STAT signaling and promotes muscle protein degradation through FOXO-mediated upregulation of ubiquitin ligases. Preclinical studies have demonstrated that interventions targeting inflammatory pathways, including COX-2 inhibition, can attenuate muscle atrophy in metabolic disease models [44,45]. Moreover, the PGE2 signaling network, which mediates diverse inflammatory effects through EP receptor subtypes, has emerged as a potential therapeutic target for conditions characterized by chronic inflammation and tissue degeneration [46]. These findings suggest that individuals identified as high-risk by UHR-BRI screening might benefit from targeted anti-inflammatory or immunomodulatory interventions, although this hypothesis requires prospective validation.

The OBS, a composite indicator integrating dietary nutrients and lifestyle factors, reflects an increased burden of oxidative stress when elevated. The present study identified a significant association between OBS and sarcopenia, a finding that has been corroborated by previous research [47]. Moreover, mediation analysis revealed that OBS partially mediated the effect of UHR-BRI on sarcopenia, confirming the key role of oxidative stress in the pathogenesis of sarcopenia. The mechanistic cascade linking UHR-BRI elevation to sarcopenia likely involves multiple interconnected pathways. Elevated uric acid activates NADPH oxidase and induces endothelial dysfunction, while reduced HDL-C impairs reverse cholesterol transport and exacerbates chronic low-grade inflammation. These processes converge to generate excessive ROS. ROS directly damage cell membrane lipids, proteins, and mitochondrial DNA, resulting in insufficient ATP production to meet the energy demands of muscle contraction and repair, ultimately causing muscle atrophy [48]. Concurrently, ROS can activate the ubiquitin-proteasome system and autophagy-lysosome pathways through forkhead box O (FOXO) and nuclear factor‑κB (NF-κB) signaling [49,50], leading to enhanced catabolism of muscle proteins and contributing to the development of sarcopenia. Furthermore, oxidative stress can trigger endoplasmic reticulum stress and the unfolded protein response, which have been increasingly recognized as key drivers of skeletal muscle atrophy in metabolic diseases [51]. These findings suggest that intervention strategies targeting oxidative stress may offer new approaches for the prevention and treatment of sarcopenia.

Beyond direct oxidative damage, the inflammatory microenvironment shaped by immune cell polarization may play a pivotal role in UHR-BRI-associated muscle loss. Macrophages, as key orchestrators of muscle repair and degeneration, exhibit a dynamic M1-to-M2 phenotypic switch during muscle homeostasis [52,53]. Persistent oxidative stress skews this balance toward sustained M1 dominance, promoting the secretion of pro-inflammatory cytokines such as TNF-α and IL-6, which activate NF-κB and JAK/STAT signaling and accelerate muscle protein breakdown. Notably, the cGAS-STING pathway has been identified as a critical mediator of sterile inflammation in skeletal muscle, with aberrant activation driving chronic inflammation and muscle atrophy [54]. The mediating role of OBS observed in our study may therefore reflect not only direct oxidative injury but also the broader immunometabolic reprogramming that links metabolic dysregulation to muscle degeneration.

Notably, the present study found that UHR-BRI was not only independently associated with sarcopenia but also the comorbidities of sarcopenia with multiple chronic diseases, including obesity, diabetes, cardiovascular disease and so on. This finding suggests that, as a multiple composite risk index, UHR-BRI reflects underlying disturbances in purine metabolism, lipid metabolism, and central obesity, which may represent a shared pathophysiological basis for the coexistence of sarcopenia and its comorbidities [55,56]. Furthermore, individuals with sarcopenia comorbidities are at risk of entering a vicious cycle in which muscle loss and chronic diseases mutually exacerbate each other. UHR-BRI, as a readily available indicator that can be calculated from routine blood tests and physical examinations, facilitates the early identification of such high-risk populations. This provides a reliable tool for the early screening and precision intervention of sarcopenia and its comorbidities, carrying important public health implications.

The present study has several notable strengths. First, we combined UHR with BRI to construct a multiple composite risk index, which showed stronger associations with sarcopenia. Second, our study was based on two large, nationally representative databases, enhancing the reliability of the findings. Third, in addition to sarcopenia alone, we also analyzed individuals with sarcopenia comorbid with multiple chronic diseases, making the findings more clinically applicable. Fourth, we employed multi‑method analytical strategy ranging from traditional regression analysis to machine learning and SHAP-based interpretability analysis, and conducted multiple sensitivity analyses to ensure the robustness of the main conclusions.

Nevertheless, several limitations should be acknowledged. First, a key limitation of this study is the inconsistent diagnostic criteria for sarcopenia. Sarcopenia was defined using AWGS-based criteria in CHARLS but FNIH muscle mass criteria in NHANES. These definitions identify distinct clinical phenotypes, which may partly account for the differences in effect sizes and discriminative performance observed between cohorts. Second, due to the cross‑sectional nature of baseline measurements in NHANES, in which UHR-BRI, OBS, and sarcopenia indicators were assessed simultaneously, temporal causality between UHR-BRI and sarcopenia cannot be established, nor can the mediating role of OBS in this association. Prospective studies are warranted to validate these findings. Third, despite adjusting for confounding factors, residual confounding cannot be completely ruled out. Additionally, in the CHARLS cohort, muscle mass assessment did not employ gold‑standard imaging techniques such as DXA, which may have introduced measurement error and misclassification of sarcopenia.

## Conclusion

In this multi-cohort study, we demonstrate that the UHR-BRI, which synergistically captures disturbances in purine metabolism, lipid metabolism, and obesity status, is significantly associated with sarcopenia and its comorbidities, with oxidative balance score identified as a potential mediator of this association warranting prospective validation.

## Supplementary Material

Supplementary Materials.docx

## Data Availability

The data that support the findings of this study are derived from two publicly available databases: the CHARLS (https://charls.pku.edu.cn/) and the NHANES (https://www.cdc.gov/nchs/nhanes/). The analytic code and derived datasets generated during this study are available from the corresponding author upon reasonable request.
