# Supplementary material for: The multiple composite risk index estimates the risks of sarcopenia and comorbidities: unveiling the role of oxidative balance score
Source: Ann Med. 2026 Sep 2;58(1):2723681. doi: 10.1080/07853890.2026.2723681 (PMC13539785; doi:10.1080/07853890.2026.2723681)
Supplement: Supplementary file 1 [file IANN_A_2723681_SM0695.docx]

**Supplementary Materials**

Table S1. Baseline characteristics according to the combination of the UHR and BRI in the CHARLS cohort

| Variables | Type1 | Type2 | Type3 | Type4 | Type5 | Type6 | Type7 | Type8 | P-value |
| --- | --- | --- | --- | --- | --- | --- | --- | --- | --- |
|  | (N=1338) | (N=1141) | (N=962) | (N=842) | (N=798) | (N=993) | (N=1172) | (N=1291) |  |
| Age, years, mean (SD) | 58.9 (9.2) | 58.6 (9.1) | 58.3 (9.1) | 60.7 (9.8) | 59.7 (9.3) | 59.5 (9.3) | 59.4 (9.3) | 60.8 (9.3) | <0.001 |
| Gender, n (%) |  |  |  |  |  |  |  |  | <0.001 |
| Male | 585(43.7%) | 692(60.6%) | 722(75.1%) | 765(90.9%) | 125(15.7%) | 296(29.8%) | 491(41.9%) | 858(66.5%) |  |
| Female | 753(56.3%) | 449(39.4%) | 240(24.9%) | 77(9.1%) | 673(84.3%) | 697(70.2%) | 681(58.1%) | 433(33.5%) |  |
| Marital Status, n (%) |  |  |  |  |  |  |  |  | <0.001 |
| Single | 144(10.8%) | 150(13.1%) | 117(12.2%) | 150(17.8%) | 95(11.9%) | 104(10.5%) | 115(9.8%) | 165(12.8%) |  |
| Married | 1194(89.2%) | 991(86.9%) | 845(87.8%) | 692(82.2%) | 703(88.1%) | 889(89.5%) | 1057(90.2%) | 1126(87.2%) |  |
| Educational Level, n (%) |  |  |  |  |  |  |  |  | <0.001 |
| Less than high school | 953(71.2%) | 818(71.7%) | 701(72.9%) | 681(80.9%) | 512(64.2%) | 645(65%) | 733(62.5%) | 932(72.2%) |  |
| High school | 354(26.5%) | 297(26%) | 241(25.1%) | 150(17.8%) | 260(32.6%) | 301(30.3%) | 376(32.1%) | 316(24.5%) |  |
| College or above | 31(2.3%) | 26(2.3%) | 20(2.1%) | 11(1.3%) | 26(3.3%) | 47(4.7%) | 63(5.4%) | 43(3.3%) |  |
| Cholesterol, mg/dl, mean (SD) | 188.7 (35.3) | 194.4 (35.9) | 199.2 (36.0) | 203.3 (36.5) | 180.5 (38.2) | 186.6 (37.5) | 194.1 (39.0) | 200.6 (41.1) | <0.001 |
| TG, mg/dl, mean (SD) | 87.4 (43.8) | 96.0 (48.5) | 105.9 (49.8) | 114.3 (53.8) | 122.6 (78.7) | 148.6 (100.9) | 169.8 (121.2) | 195.5 (128.4) | <0.001 |
| FBG, mg/dl, mean (SD) | 103.6 (30.6) | 105.6 (35.3) | 109.4 (43.2) | 112.1 (38.5) | 104.5 (27.9) | 108.7 (29.9) | 115.7 (39.3) | 119.9 (41.2) | <0.001 |
| LDL, mg/dl, mean (SD) | 110.6 (31.6) | 116.5 (32.0) | 122.1 (32.2) | 126.8 (32.6) | 109.7 (32.9) | 112.7 (34.8) | 117.3 (38.0) | 119.3 (39.9) | <0.001 |
| BUN, mg/dl, mean (SD) | 16.1 (4.7) | 15.6 (4.4) | 15.1 (4.4) | 15.1 (4.2) | 16.2 (4.7) | 16.3 (5.3) | 15.9 (4.3) | 15.8 (4.5) | <0.001 |
| Cr, mg/dl, mean (SD) | 0.8 (0.2) | 0.7 (0.2) | 0.7 (0.4) | 0.7 (0.1) | 0.9 (0.2) | 0.9 (0.3) | 0.8 (0.3) | 0.8 (0.2) | <0.001 |
| CRP, mg/l, mean (SD) | 2.0 (5.3) | 2.0 (6.5) | 2.6 (8.6) | 2.3 (5.7) | 3.4 (10.9) | 2.9 (8.6) | 3.0 (7.5) | 3.6 (7.3) |  |
| Smoking, n (%) |  |  |  |  |  |  |  |  | <0.001 |
| No | 657(49.1%) | 387(33.9%) | 210(21.8%) | 99(11.8%) | 543(68.0%) | 558(56.2%) | 539(46.0%) | 365(28.3%) |  |
| Yes | 681(50.9%) | 754(66.1%) | 752(78.2%) | 743(88.2%) | 255(32.0%) | 435(43.8%) | 633(54.0%) | 926(71.7%) |  |
| Alcohol Use, n (%) |  |  |  |  |  |  |  |  | <0.001 |
| No | 541(40.4%) | 372(32.6%) | 241(25.1%) | 125(14.8%) | 371(46.5%) | 422(42.5%) | 421(35.9%) | 303(23.5%) |  |
| Yes | 797(59.6%) | 769(67.4%) | 721(74.9%) | 717(85.2%) | 427(53.5%) | 571(57.5%) | 751(64.1%) | 988(76.5%) |  |
| Obesity, n (%) |  |  |  |  |  |  |  |  | <0.001 |
| No | 1334(99.7%) | 1134(99.4%) | 928(96.5%) | 586(69.6%) | 795(99.6%) | 983(99.0%) | 1103(94.1%) | 698(54.1%) |  |
| Yes | 4(0.3%) | 7(0.6%) | 34(3.5%) | 256(30.4%) | 3(0.4%) | 10(1.0%) | 69(5.9%) | 593(45.9%) |  |
| Diabetes, n (%) |  |  |  |  |  |  |  |  | <0.001 |
| No | 1310(97.9%) | 1095(96.0%) | 914(95.0%) | 771(91.6%) | 780(97.7%) | 952(95.9%) | 1087(92.7%) | 1143(88.5%) |  |
| Yes | 28(2.1%) | 46(4.0%) | 48(5.0%) | 71(8.4%) | 18(2.3%) | 41(4.1%) | 85(7.3%) | 148(11.5%) |  |
| Kidney Diseases, n (%) |  |  |  |  |  |  |  |  | 0.059 |
| No | 1231(92.0%) | 1076(94.3%) | 890(92.5%) | 802(95.2%) | 742(93.0%) | 921(92.7%) | 1095(93.4%) | 1215(94.1%) |  |
| Yes | 107(8.0%) | 65(5.7%) | 72(7.5%) | 40(4.8%) | 56(7.0%) | 72(7.3%) | 77(6.6%) | 76(5.9%) |  |
| Cancers, n (%) |  |  |  |  |  |  |  |  | 0.582 |
| No | 1327(99.2%) | 1133(99.3%) | 954(99.2%) | 830(98.6%) | 793(99.4%) | 987(99.4%) | 1160(99%) | 1277(98.9%) |  |
| Yes | 11(0.8%) | 8(0.7%) | 8(0.8%) | 12(1.4%) | 5(0.6%) | 6(0.6%) | 12(1%) | 14(1.1%) |  |
| Stroke, n (%) |  |  |  |  |  |  |  |  | <0.001 |
| No | 1322(98.8%) | 1131(99.1%) | 947(98.4%) | 825(98.0%) | 783(98.1%) | 975(98.2%) | 1145(97.7%) | 1241(96.1%) |  |
| Yes | 16(1.2%) | 10(0.9%) | 15(1.6%) | 17(2.0%) | 15(1.9%) | 18(1.8%) | 27(2.3%) | 50(3.9%) |  |
| CVD, n (%) |  |  |  |  |  |  |  |  | <0.001 |
| No | 1232(92.1%) | 1030(90.3%) | 841(87.4%) | 727(86.3%) | 726(91.0%) | 889(89.5%) | 1025(87.5%) | 1065(82.5%) |  |
| Yes | 106(7.9%) | 111(9.7%) | 121(12.6%) | 115(13.7%) | 72(9.0%) | 104(10.5%) | 147(12.5%) | 226(17.5%) |  |
| Sarcopenia, n (%) |  |  |  |  |  |  |  |  | <0.001 |
| No | 1296(96.9%) | 1027(90.0%) | 814(84.6%) | 611(72.6%) | 779(97.6%) | 925(93.2%) | 1015(86.6%) | 988(76.5%) |  |
| Yes | 42(3.1%) | 114(10.0%) | 148(15.4%) | 231(27.4%) | 19(2.4%) | 68(6.8%) | 157(13.4%) | 303(23.5%) |  |

UHR, uric acid to high-density lipoprotein cholesterol ratio; BRI, body roundness index; TG, triglyceride; FBG, fasting blood glucose; LDL, low density lipoprotein; BUN, blood urea nitrogen; Cr, creatinine; CRP, C-reactive protein; CVD, cardiovascular disease; SD, standard deviation; CHARLS, China Health and Retirement Longitudinal Study.

Table S2. Baseline characteristics according to the combination of the UHR and BRI in the NHANES cohort

| Variables | Type1 | Type2 | Type3 | Type4 | Type5 | Type6 | Type7 | Type8 | P-value |
| --- | --- | --- | --- | --- | --- | --- | --- | --- | --- |
|  | (N=1522) | (N=1152) | (N=907) | (N=757) | (N=606) | (N=975) | (N=1220) | (N=1371) |  |
| Age, years, mean (SD) | 34.3 (11.2) | 40.2 (11.4) | 42.2 (11.3) | 42.9 (10.7) | 32.0 (10.3) | 38.8 (11.0) | 41.0 (10.8) | 40.8 (11.1) | <0.001 |
| Gender, n (%) |  |  |  |  |  |  |  |  | <0.001 |
| Male | 600(39.4%) | 338(29.3%) | 208(22.9%) | 76(10.0%) | 556(91.7%) | 857(87.9%) | 918(75.2%) | 680(49.6%) |  |
| Female | 922(60.6%) | 814(70.7%) | 699(77.1%) | 681(90.0%) | 50(8.3%) | 118(12.1%) | 302(24.8%) | 691(50.4%) |  |
| Race, n (%) |  |  |  |  |  |  |  |  | <0.001 |
| Mexican American | 102(6.7%) | 156(13.5%) | 178(19.6%) | 162(21.4%) | 29(4.8%) | 112(11.5%) | 237(19.4%) | 267(19.5%) |  |
| Other Hispanic | 116(7.6%) | 122(10.6%) | 91(10.0%) | 82(10.8%) | 38(6.3%) | 97(9.9%) | 147(12%) | 148(10.8%) |  |
| Non-Hispanic White | 587(38.6%) | 440(38.2%) | 313(34.5%) | 247(32.6%) | 252(41.6%) | 356(36.5%) | 455(37.3%) | 538(39.2%) |  |
| Non-Hispanic Black | 352(23.1%) | 216(18.8%) | 200(22.1%) | 221(29.2%) | 118(19.5%) | 140(14.4%) | 180(14.8%) | 297(21.7%) |  |
| Others | 365(24.0%) | 218(18.9%) | 125(13.8%) | 45(5.9%) | 169(27.9%) | 270(27.7%) | 201(16.5%) | 121(8.8%) |  |
| Marital Status, n (%) |  |  |  |  |  |  |  |  | <0.001 |
| Single | 774(50.9%) | 423(36.7%) | 361(39.8%) | 332(43.9%) | 308(50.8%) | 326(33.4%) | 407(33.4%) | 516(37.6%) |  |
| Married | 748(49.1%) | 729(63.3%) | 546(60.2%) | 425(56.1%) | 298(49.2%) | 649(66.6%) | 813(66.6%) | 855(62.4%) |  |
| Educational Level, n (%) |  |  |  |  |  |  |  |  | <0.001 |
| Less than high school | 170(11.2%) | 173(15.0%) | 160(17.6%) | 159(21.0%) | 83(13.7%) | 155(15.9%) | 244(20.0%) | 259(18.9%) |  |
| High school | 287(18.9%) | 221(19.2%) | 195(21.5%) | 172(22.7%) | 130(21.5%) | 205(21.0%) | 292(23.9%) | 360(26.3%) |  |
| College or above | 1065(70.0%) | 758(65.8%) | 552(60.9%) | 426(56.3%) | 393(64.9%) | 615(63.1%) | 684(56.1%) | 752(54.9%) |  |
| PIR, mean (SD) | 2.6 (1.7) | 2.7 (1.7) | 2.6 (1.7) | 2.3 (1.5) | 2.5 (1.7) | 2.8 (1.7) | 2.5 (1.6) | 2.2 (1.5) | <0.001 |
| Cholesterol, mg/dl, mean (SD) | 179.3 (35.2) | 190.9 (37.4) | 195.3 (39.3) | 195.4 (37.8) | 175.0 (38.4) | 193.9 (40.4) | 198.3 (41.0) | 192.6 (40.8) | <0.001 |
| TG, mg/dl, mean (SD) | 101.0 (106.9) | 128.9 (147.8) | 147.5 (159.6) | 134.2 (149.9) | 151.4 (138.2) | 198.3 (179.5) | 218.5 (198.2) | 209.0 (179.9) | <0.001 |
| FBG, mg/dl, mean (SD) | 97.6 (24.3) | 101.5 (30.7) | 105.3 (31.1) | 114.3 (45.5) | 99.9 (22.1) | 105.1 (27.4) | 109.3 (30.6) | 119.4 (44.6) | <0.001 |
| LDL, mg/dl, mean (SD) | 94.9 (31.7) | 104.3 (33.8) | 108.8 (34.9) | 113.4 (33.4) | 99.1 (32.0) | 112.2 (36.3) | 114.6 (37.7) | 110.8 (36.5) | <0.001 |
| WBC, 10^3^/uL, mean (SD) | 6.6 (1.9) | 6.8 (1.9) | 7.3 (2.1) | 7.7 (2.3) | 6.9 (2.1) | 7.1 (2.0) | 7.6 (2.1) | 8.3 (2.3) | <0.001 |
| RBC, 10^6^/uL, mean (SD) | 4.6 (0.5) | 4.6 (0.4) | 4.6 (0.4) | 4.6 (0.4) | 5.0 (0.4) | 5.0 (0.5) | 4.9 (0.5) | 4.9 (0.5) | <0.001 |
| Hb, g/dl, mean (SD) | 13.9 (1.4) | 13.6 (1.5) | 13.5 (1.5) | 13.3 (1.4) | 14.9 (1.1) | 14.9 (1.3) | 14.8 (1.4) | 14.2 (1.6) | <0.001 |
| PLT,10^3^/uL, mean (SD) | 233.1 (52.2) | 242.3 (58.6) | 254.6 (58.9) | 267.6 (69.9) | 227.2 (51.6) | 234.8 (56.5) | 242.0 (58.2) | 257.8 (65.5) | <0.001 |
| ALT, U/L, mean (SD) | 20.5 (15.3) | 22.5 (24.4) | 22.6 (14.0) | 22.6 (17.4) | 23.8 (15.6) | 28.5 (18.3) | 31.5 (22.7) | 32.1 (23.5) | <0.001 |
| AST, U/L, mean (SD) | 33.0 (24.2) | 32.8 (24.7) | 35.0 (27.2) | 35.9 (31.7) | 34.0 (33.9) | 36.1 (26.1) | 38.5 (35.9) | 41.1 (36.3) | <0.001 |
| BUN, mg/dl, mean (SD) | 11.9 (3.8) | 12.2 (4.0) | 12.1 (4.3) | 11.7 (3.8) | 12.8 (4.5) | 13.4 (4.1) | 13.3 (4.9) | 12.6 (4.8) | <0.001 |
| LDH, U/L, mean (SD) | 122.1 (24.4) | 127.1 (27.0) | 132.1 (29.9) | 134.2 (29.5) | 125.6 (34.9) | 128.2 (26.1) | 132.8 (33.1) | 138.0 (31.0) | <0.001 |
| TBil, mg/dl, mean (SD) | 0.6 (0.3) | 0.6 (0.3) | 0.5 (0.3) | 0.5 (0.2) | 0.8 (0.4) | 0.7 (0.3) | 0.6 (0.3) | 0.5 (0.3) | <0.001 |
| Cr, mg/dl, mean (SD) | 0.8 (0.2) | 0.8 (0.3) | 0.8 (0.6) | 0.7 (0.3) | 1.0 (0.3) | 1.0 (0.2) | 0.9 (0.5) | 0.9 (0.2) | <0.001 |
| OBS, mean (SD) | 8.2 (1.3) | 7.8 (1.3) | 7.0 (1.5) | 6.3 (1.2) | 8.3 (1.5) | 7.7 (1.4) | 6.9 (1.3) | 6.3 (1.3) | <0.001 |
| Smoking, n (%) |  |  |  |  |  |  |  |  | <0.001 |
| No | 978(64.3%) | 746(64.8%) | 585(64.5%) | 495(65.4%) | 346(57.1%) | 549(56.3%) | 667(54.7%) | 768(56.0%) |  |
| Yes | 544(35.7%) | 406(35.2%) | 322(35.5%) | 262(34.6%) | 260(42.9%) | 426(43.7%) | 553(45.3%) | 603(44.0%) |  |
| Alcohol Use, n (%) |  |  |  |  |  |  |  |  | <0.001 |
| No | 452(29.7%) | 289(25.1%) | 218(24.0%) | 139(18.4%) | 136(22.4%) | 183(18.8%) | 199(16.3%) | 213(15.5%) |  |
| Yes | 1070(70.3%) | 863(74.9%) | 689(76.0%) | 618(81.6%) | 470(77.6%) | 792(81.2%) | 1021(83.7%) | 1158(84.5%) |  |
| Physical activity, n (%) |  |  |  |  |  |  |  |  | 0.840 |
| Insufficient | 244(16.0%) | 188(16.3%) | 150(16.5%) | 114(15.1%) | 95(15.7%) | 157(16.1%) | 207(17%) | 208(15.2%) |  |
| Sufficient | 259(17.0%) | 208(18.1%) | 168(18.5%) | 148(19.6%) | 104(17.2%) | 194(19.9%) | 202(16.6%) | 250(18.2%) |  |
| Exceeding | 1019(67.0%) | 756(65.6%) | 589(64.9%) | 495(65.4%) | 407(67.2%) | 624(64.0%) | 811(66.5%) | 913(66.6%) |  |
| Obesity, n (%) |  |  |  |  |  |  |  |  | <0.001 |
| No | 1522(100.0%) | 1112(96.5%) | 544(60.0%) | 33(4.4%) | 606(100.0%) | 916(93.9%) | 587(48.1%) | 30(2.2%) |  |
| Yes | 0(0.0%) | 40(3.5%) | 363(40.0%) | 724(95.6%) | 0(0.0%) | 59(6.1%) | 633(51.9%) | 1341(97.8%) |  |
| Diabetes, n (%) |  |  |  |  |  |  |  |  | <0.001 |
| No | 1502(98.7%) | 1106(96.0%) | 838(92.4%) | 652(86.1%) | 591(97.5%) | 929(95.3%) | 1129(92.5%) | 1142(83.3%) |  |
| Yes | 20(1.3%) | 46(4.0%) | 69(7.6%) | 105(13.9%) | 15(2.5%) | 46(4.7%) | 91(7.5%) | 229(16.7%) |  |
| Kidney Diseases, n (%) |  |  |  |  |  |  |  |  | 0.039 |
| No | 1500(98.6%) | 1138(98.8%) | 887(97.8%) | 745(98.4%) | 601(99.2%) | 959(98.4%) | 1192(97.7%) | 1335(97.4%) |  |
| Yes | 22(1.4%) | 14(1.2%) | 20(2.2%) | 12(1.6%) | 5(0.8%) | 16(1.6%) | 28(2.3%) | 36(2.6%) |  |
| Cancers, n (%) |  |  |  |  |  |  |  |  | <0.001 |
| No | 1468(96.5%) | 1104(95.8%) | 869(95.8%) | 716(94.6%) | 597(98.5%) | 949(97.3%) | 1182(96.9%) | 1302(95%) |  |
| Yes | 54(3.5%) | 48(4.2%) | 38(4.2%) | 41(5.4%) | 9(1.5%) | 26(2.7%) | 38(3.1%) | 69(5.0%) |  |
| Stroke, n (%) |  |  |  |  |  |  |  |  | <0.001 |
| No | 1510(99.2%) | 1138(98.8%) | 892(98.3%) | 735(97.1%) | 604(99.7%) | 966(99.1%) | 1195(98%) | 1341(97.8%) |  |
| Yes | 12(0.8%) | 14(1.2%) | 15(1.7%) | 22(2.9%) | 2(0.3%) | 9(0.9%) | 25(2.0%) | 30(2.2%) |  |
| CVD, n (%) |  |  |  |  |  |  |  |  | <0.001 |
| No | 1495(98.2%) | 1119(97.1%) | 875(96.5%) | 710(93.8%) | 595(98.2%) | 947(97.1%) | 1162(95.2%) | 1282(93.5%) |  |
| Yes | 27(1.8%) | 33(2.9%) | 32(3.5%) | 47(6.2%) | 11(1.8%) | 28(2.9%) | 58(4.8%) | 89(6.5%) |  |
|  |  |  |  |  |  |  |  |  |  |
| Sarcopenia, n (%) |  |  |  |  |  |  |  |  | <0.001 |
| No | 1512(99.3%) | 1129(98.0%) | 822(90.6%) | 579(76.5%) | 604(99.7%) | 944(96.8%) | 1119(91.7%) | 1085(79.1%) |  |
| Yes | 10(0.7%) | 23(2.0%) | 85(9.4%) | 178(23.5%) | 2(0.3%) | 31(3.2%) | 101(8.3%) | 286(20.9%) |  |

UHR, uric acid to high-density lipoprotein cholesterol ratio; BRI, body roundness index; PIR, poverty income ratio; TG, triglyceride; FBG, fasting blood glucose; LDL, low density lipoprotein; WBC, white blood cell; RBC, red blood cell; Hb, hemoglobin; PLT, platelet; ALT, alanine aminotransferase; AST, aspartate aminotransferase; BUN, blood urea nitrogen; LDH, lactate dehydrogenase; TBil, total bilirubin; Cr, creatinine; OBS, oxidative balance score; CVD, cardiovascular disease; SD, standard deviation; NHANES, National Health and Nutrition Examination Survey.

Table S3. Baseline characteristics of the participants according to the UHR-BRI quartiles in the NHANES cohort

| Variables | Overall | Q1 | Q2 | Q3 | Q4 | P-value |
| --- | --- | --- | --- | --- | --- | --- |
|  | (N=8510) | (N=2134) | (N=2124) | (N=2126) | (N=2126) |  |
| Age, years, mean (SD) | 39.1 (11.5) | 35.7 (11.6) | 39.1 (11.8) | 41.0 (11.0) | 40.5 (11.1) | <0.001 |
| Gender, n (%) |  |  |  |  |  | <0.001 |
| Male | 4233(49.7%) | 719(33.7%) | 1018(47.9%) | 1211(57.0%) | 1285(60.4%) |  |
| Female | 4277(50.3%) | 1415(66.3%) | 1106(52.1%) | 915(43.0%) | 841(39.6%) |  |
| Race, n (%) |  |  |  |  |  | <0.001 |
| Mexican American | 1243(14.6%) | 192(9.0%) | 278(13.1%) | 377(17.7%) | 396(18.6%) |  |
| Other Hispanic | 841(9.9%) | 174(8.2%) | 202(9.5%) | 224(10.5%) | 241(11.3%) |  |
| Non-Hispanic White | 3188(37.5%) | 828(38.8%) | 785(37%) | 756(35.6%) | 819(38.5%) |  |
| Non-Hispanic Black | 1724(20.3%) | 486(22.8%) | 432(20.3%) | 400(18.8%) | 406(19.1%) |  |
| Others | 1514(17.8%) | 454(21.3%) | 427(20.1%) | 369(17.4%) | 264(12.4%) |  |
| Marital Status, n (%) |  |  |  |  |  | <0.001 |
| Single | 3447(40.5%) | 1023(47.9%) | 876(41.2%) | 778(36.6%) | 770(36.2%) |  |
| Married | 5063(59.5%) | 1111(52.1%) | 1248(58.8%) | 1348(63.4%) | 1356(63.8%) |  |
| Educational Level, n (%) |  |  |  |  |  | <0.001 |
| Less than high school | 1403(16.5%) | 251(11.8%) | 339(16.0%) | 405(19.0%) | 408(19.2%) |  |
| High school | 1862(21.9%) | 404(18.9%) | 444(20.9%) | 470(22.1%) | 544(25.6%) |  |
| College or above | 5245(61.6%) | 1479(69.3%) | 1341(63.1%) | 1251(58.8%) | 1174(55.2%) |  |
| PIR, mean (SD) | 2.5 (1.7) | 2.6 (1.7) | 2.6 (1.7) | 2.6 (1.7) | 2.3 (1.6) | <0.001 |
| Cholesterol, mg/dl, mean (SD) | 190.2 (39.5) | 181.6 (35.8) | 190.7 (40.1) | 195.1 (40.0) | 193.5 (40.4) | <0.001 |
| TG, mg/dl, mean (SD) | 161.7 (165.7) | 96.4 (106.8) | 154.9 (161.6) | 185.6 (173.2) | 209.9 (187.7) | <0.001 |
| FBG, mg/dl, mean (SD) | 106.7 (33.9) | 97.7 (23.0) | 104.0 (32.1) | 108.6 (33.7) | 116.3 (41.6) | <0.001 |
| LDL, mg/dl, mean (SD) | 107.0 (35.4) | 97.4 (32.0) | 105.8 (35.2) | 111.2 (35.1) | 113.5 (37.0) | <0.001 |
| WBC,10^3^/uL, mean (SD) | 7.3 (2.2) | 6.6 (1.9) | 7.1 (2.1) | 7.4 (2.1) | 8.1 (2.2) | <0.001 |
| RBC,10^6^/uL, mean (SD) | 4.7 (0.5) | 4.6 (0.4) | 4.7 (0.5) | 4.8 (0.5) | 4.9 (0.5) | <0.001 |
| Hb, g/dl, mean (SD) | 14.1 (1.5) | 13.7 (1.5) | 14.1 (1.5) | 14.3 (1.5) | 14.4 (1.6) | <0.001 |
| PLT,10^3^/uL, mean (SD) | 244.7 (60.2) | 236.1 (55.6) | 242.0 (57.7) | 248.0 (61.8) | 252.9 (64.1) | <0.001 |
| ALT, U/L, mean (SD) | 25.8 (20.2) | 20.4 (15.2) | 23.5 (20.5) | 26.7 (19.1) | 32.5 (23.3) | <0.001 |
| AST, U/L, mean (SD) | 36.0 (30.3) | 33.3 (27.5) | 33.7 (26.2) | 37.2 (33.4) | 39.7 (33.2) | <0.001 |
| BUN, mg/dl, mean (SD) | 12.5 (4.3) | 11.9 (3.9) | 12.4 (4.1) | 12.8 (4.3) | 12.9 (4.9) | <0.001 |
| LDH, U/L, mean (SD) | 130.0 (29.7) | 123.7 (26.5) | 128.1 (28.0) | 132.0 (31.0) | 136.0 (31.6) | <0.001 |
| TBil, mg/dl, mean (SD) | 0.6 (0.3) | 0.6 (0.3) | 0.6 (0.3) | 0.6 (0.3) | 0.6 (0.3) | <0.001 |
| Cr, mg/dl, mean (SD) | 0.9 (0.4) | 0.8 (0.3) | 0.8 (0.3) | 0.9 (0.5) | 0.9 (0.4) | <0.001 |
| OBS, mean (SD) | 7.3 (1.5) | 8.1 (1.4) | 7.6 (1.5) | 7.1 (1.4) | 6.5 (1.4) | <0.001 |
| Smoking, n (%) |  |  |  |  |  | <0.001 |
| No | 5134(60.3%) | 1379(64.6%) | 1309(61.6%) | 1253(58.9%) | 1193(56.1%) |  |
| Yes | 3376(39.7%) | 755(35.4%) | 815(38.4%) | 873(41.1%) | 933(43.9%) |  |
| Alcohol Use, n (%) |  |  |  |  |  | <0.001 |
| No | 1829(21.5%) | 540(25.3%) | 452(21.3%) | 446(21.0%) | 391(18.4%) |  |
| Yes | 6681(78.5%) | 1594(74.7%) | 1672(78.7%) | 1680(79.0%) | 1735(81.6%) |  |
| Physical activity, n (%) |  |  |  |  |  | <0.001 |
| Insufficient | 1363(16.0%) | 337(15.8%) | 348(16.4%) | 344(16.2%) | 334(15.7%) |  |
| Sufficient | 1533(18.0%) | 370(17.3%) | 388(18.3%) | 380(17.9%) | 395(18.6%) |  |
| Exceeding | 5614(66.0%) | 1427(66.9%) | 1388(65.3%) | 1402(65.9%) | 1397(65.7%) |  |
| Obesity, n (%) |  |  |  |  |  | <0.001 |
| No | 5350(62.9%) | 2095(98.2%) | 1735(81.7%) | 1147(54.0%) | 373(17.5%) |  |
| Yes | 3160(37.1%) | 39(1.8%) | 389(18.3%) | 979(46.0%) | 1753(82.5%) |  |
| Diabetes, n (%) |  |  |  |  |  | <0.001 |
| No | 7889(92.7%) | 2092(98.0%) | 2015(94.9%) | 1949(91.7%) | 1833(86.2%) |  |
| Yes | 621(7.3%) | 42(2.0%) | 109(5.1%) | 177(8.3%) | 293(13.8%) |  |
| Kidney Diseases, n (%) |  |  |  |  |  | 0.027 |
| No | 8357(98.2%) | 2105(98.6%) | 2092(98.5%) | 2087(98.2%) | 2073(97.5%) |  |
| Yes | 153(1.8%) | 29(1.4%) | 32(1.5%) | 39(1.8%) | 53(2.5%) |  |
| Cancers, n (%) |  |  |  |  |  | 0.414 |
| No | 8187(96.2%) | 2054(96.3%) | 2054(96.7%) | 2044(96.1%) | 2035(95.7%) |  |
| Yes | 323(3.8%) | 80(3.7%) | 70(3.3%) | 82(3.9%) | 91(4.3%) |  |
| Stroke, n (%) |  |  |  |  |  | 0.001 |
| No | 8381(98.5%) | 2118(99.3%) | 2096(98.7%) | 2088(98.2%) | 2079(97.8%) |  |
| Yes | 129(1.5%) | 16(0.7%) | 28(1.3%) | 38(1.8%) | 47(2.2%) |  |
| CVD, n (%) |  |  |  |  |  | <0.001 |
| No | 8185(96.2%) | 2094(98.1%) | 2059(96.9%) | 2038(95.9%) | 1994(93.8%) |  |
| Yes | 325(3.8%) | 40(1.9%) | 65(3.1%) | 88(4.1%) | 132(6.2%) |  |
| Sarcopenia, n (%) |  |  |  |  |  | <0.001 |
| No | 7794(91.6%) | 2109(98.8%) | 2021(95.2%) | 1919(90.3%) | 1745(82.1%) |  |
| Yes | 716(8.4%) | 25(1.2%) | 103(4.8%) | 207(9.7%) | 381(17.9%) |  |

UHR, uric acid to high-density lipoprotein cholesterol ratio; BRI, body roundness index; PIR, poverty income ratio; TG, triglyceride; FBG, fasting blood glucose; LDL, low density lipoprotein; WBC, white blood cell; RBC, red blood cell; Hb, hemoglobin; PLT, platelet; ALT, alanine aminotransferase; AST, aspartate aminotransferase; BUN, blood urea nitrogen; LDH, lactate dehydrogenase; TBil, total bilirubin; Cr, creatinine; OBS, oxidative balance score; CVD, cardiovascular disease; SD, standard deviation; NHANES, National Health and Nutrition Examination Survey.

Table S4. Baseline characteristics of the study population stratified by sarcopenia status in the CHARLS cohort

| Variables | Sarcopenia | | P-value |
| --- | --- | --- | --- |
|  | No(N=7455) | Yes(N=1082) |  |
| Age, years, mean (SD) | 58.4 (8.8) | 66.6 (9.7) | <0.001 |
| Gender, n (%) |  |  | <0.001 |
| Male | 3893(52.2%) | 641(59.2%) |  |
| Female | 3562(47.8%) | 441(40.8%) |  |
| Marital Status, n (%) |  |  | <0.001 |
| Single | 781(10.5%) | 259(23.9%) |  |
| Married | 6674(89.5%) | 823(76.1%) |  |
| Educational Level, n (%) |  |  | <0.001 |
| Less than high school | 5049(67.7%) | 926(85.6%) |  |
| High school | 2155(28.9%) | 140(12.9%) |  |
| College or above | 251(3.4%) | 16(1.5%) |  |
| Cholesterol, mg/dl, mean (SD) | 192.6 (37.7) | 200.3 (40.4) | <0.001 |
| TG, mg/dl, mean (SD) | 131.5 (95.4) | 130.3 (86.1) | <0.001 |
| FBG, mg/dl, mean (SD) | 109.8 (36.3) | 112.6 (39.2) | 0.022 |
| LDL, mg/dl, mean (SD) | 115.9 (34.6) | 122.2 (36.6) | <0.001 |
| BUN, mg/dl, mean (SD) | 15.7 (4.5) | 16.3 (4.9) | <0.001 |
| Cr, mg/dl, mean (SD) | 0.8 (0.2) | 0.8 (0.2) | 0.399 |
| CRP, mg/l, mean (SD) | 2.7 (7.5) | 3.2 (8.0) |  |
| Smoking, n (%) |  |  | <0.001 |
| No | 3002(40.3%) | 356(32.9%) |  |
| Yes | 4453(59.7%) | 726(67.1%) |  |
| Alcohol Use, n (%) |  |  | <0.001 |
| No | 2517(33.8%) | 279(25.8%) |  |
| Yes | 4938(66.2%) | 803(74.2%) |  |
| Obesity, n (%) |  |  | <0.001 |
| No | 6670(89.5%) | 891(82.3%) |  |
| Yes | 785(10.5%) | 191(17.7%) |  |
| Diabetes, n (%) |  |  | <0.001 |
| No | 7031(94.3%) | 1021(94.4%) |  |
| Yes | 424(5.7%) | 61(5.6%) |  |
| Kidney Diseases, n (%) |  |  | 0.591 |
| No | 6957(93.3%) | 1015(93.8%) |  |
| Yes | 498(6.7%) | 67(6.2%) |  |
| Cancers, n (%) |  |  | 0.460 |
| No | 7386(99.1%) | 1075(99.4%) |  |
| Yes | 69(0.9%) | 7(0.6%) |  |
| Stroke, n (%) |  |  | 0.091 |
| No | 7316(98.1%) | 1053(97.3%) |  |
| Yes | 139(1.9%) | 29(2.7%) |  |
| CVD, n (%) |  |  | 0.723 |
| No | 6584(88.3%) | 951(87.9%) |  |
| Yes | 871(11.7%) | 131(12.1%) |  |

UHR, uric acid to high-density lipoprotein cholesterol ratio; BRI, body roundness index; TG, triglyceride; FBG, fasting blood glucose; LDL, low density lipoprotein; BUN, blood urea nitrogen; Cr, creatinine; CRP, C-reactive protein; CVD, cardiovascular disease; SD, standard deviation; CHARLS, China Health and Retirement Longitudinal Study.

Table S5. Baseline characteristics of the study population stratified by sarcopenia status in the NHANES cohort

| Variables | Sarcopenia | | P-value |
| --- | --- | --- | --- |
|  | No (N=7794) | Yes (N=716) |  |
| Age, years, mean (SD) | 38.7 (11.5) | 43.5 (11.4) | <0.001 |
| Gender, n (%) |  |  | <0.001 |
| Male | 3877(49.7%) | 356(49.7%) |  |
| Female | 3917(50.3%) | 360(50.3%) |  |
| Race, n (%) |  |  | <0.001 |
| Mexican American | 995(12.8%) | 248(34.6%) |  |
| Other Hispanic | 731(9.4%) | 110(15.4%) |  |
| Non-Hispanic White | 2985(38.3%) | 203(28.4%) |  |
| Non-Hispanic Black | 1679(21.5%) | 45(6.3%) |  |
| Others | 1404(18.0%) | 110(15.4%) |  |
| Marital Status, n (%) |  |  | 0.023 |
| Single | 3186(40.9%) | 261(36.5%) |  |
| Married | 4608(59.1%) | 455(63.5%) |  |
| Educational Level, n (%) |  |  | <0.001 |
| Less than high school | 1196(15.3%) | 207(28.9%) |  |
| High school | 1673(21.5%) | 189(26.4%) |  |
| College or above | 4925(63.2%) | 320(44.7%) |  |
| PIR, mean (SD) | 2.6 (1.7) | 2.1 (1.6) | <0.001 |
| Cholesterol, mg/dl, mean (SD) | 189.7 (39.5) | 196.4 (39.1) | <0.001 |
| TG, mg/dl, mean (SD) | 159.4 (166.3) | 186.5 (157.1) | <0.001 |
| FBG, mg/dl, mean (SD) | 105.7 (32.6) | 117.3 (44.4) | <0.001 |
| LDL, mg/dl, mean (SD) | 106.5 (35.3) | 112.1 (36.2) | <0.001 |
| WBC,10^3^/uL, mean (SD) | 7.2 (2.2) | 8.0 (2.2) | <0.001 |
| RBC,10^6^/uL, mean (SD) | 4.7 (0.5) | 4.8 (0.5) | <0.001 |
| Hb, g/dl, mean (SD) | 14.1 (1.5) | 14.2 (1.6) | 0.336 |
| PLT,10^3^/uL, mean (SD) | 243.6 (59.8) | 257.0 (63.2) | <0.001 |
| ALT, U/L, mean (SD) | 25.4 (19.8) | 29.6 (24.4) | <0.001 |
| AST, U/L, mean (SD) | 35.4 (30.0) | 42.3 (33.1) | <0.001 |
| BUN, mg/dl, mean (SD) | 12.5 (4.3) | 12.8 (4.7) | 0.023 |
| LDH, U/L, mean (SD) | 129.5 (29.7) | 135.5 (29.7) | <0.001 |
| TBil, mg/dl, mean (SD) | 0.6 (0.3) | 0.5 (0.3) | <0.001 |
| UHR, mean (SD) | 0.09 (0.1) | 0.12 (0.1) | <0.001 |
| BRI, mean (SD) | 5.0 (2.1) | 7.7 (2.6) | <0.001 |
| Cr, mg/dl, mean (SD) | 0.9 (0.3) | 0.8 (0.4) | <0.001 |
| OBS, mean (SD) | 7.4 (1.6) | 6.7 (1.3) | <0.001 |
| Smoking, n (%) |  |  | 0.041 |
| No | 4676(60.0%) | 458(64.0%) |  |
| Yes | 3118(40.0%) | 258(36.0%) |  |
| Alcohol Use, n (%) |  |  | <0.001 |
| No | 1780(22.8%) | 49(6.9%) |  |
| Yes | 6014(77.2%) | 667(93.1%) |  |
| Physical activity, n (%) |  |  | 0.469 |
| Insufficient | 1252(16.1%) | 111(15.5%) |  |
| Sufficient | 1392(17.9%) | 141(19.7%) |  |
| Exceeding | 5150(66.1%) | 464(64.8%) |  |
| Obesity, n (%) |  |  | <0.001 |
| No | 5125(65.8%) | 225(31.4%) |  |
| Yes | 2669(34.2%) | 491(68.6%) |  |
| Diabetes, n (%) |  |  | <0.001 |
| No | 7287(93.5%) | 602(84.1%) |  |
| Yes | 507(6.5%) | 114(15.9%) |  |
| Kidney Diseases, n (%) |  |  | 0.002 |
| No | 7665(98.3%) | 692(96.6%) |  |
| Yes | 129(1.7%) | 24(3.4%) |  |
| Cancers, n (%) |  |  | 0.021 |
| No | 7510(96.4%) | 677(94.6%) |  |
| Yes | 284(3.6%) | 39(5.4%) |  |
| Stroke, n (%) |  |  | 0.006 |
| No | 7685(98.6%) | 696(97.2%) |  |
| Yes | 109(1.4%) | 20(2.8%) |  |
| CVD, n (%) |  |  | <0.001 |
| No | 7524(96.5%) | 661(92.3%) |  |
| Yes | 270(3.5%) | 55(7.7%) |  |

UHR, uric acid to high-density lipoprotein cholesterol ratio; BRI, body roundness index; PIR, poverty income ratio; TG, triglyceride; FBG, fasting blood glucose; LDL, low density lipoprotein; WBC, white blood cell; RBC, red blood cell; Hb, hemoglobin; PLT, platelet; ALT, alanine aminotransferase; AST, aspartate aminotransferase; BUN, blood urea nitrogen; LDH, lactate dehydrogenase; TBil, total bilirubin; Cr, creatinine; OBS, oxidative balance score; CVD, cardiovascular disease; SD, standard deviation; NHANES, National Health and Nutrition Examination Survey.

Table S6. Weighted logistic regression models for sarcopenia incidence by the joint of the UHR and BRI in the CHARLS cohort

| Groups | Model1 | | Model2 | | Model3 | |
| --- | --- | --- | --- | --- | --- | --- |
|  | OR (95%CI) | P-value | OR (95%CI) | P-value | OR (95%CI) | P-value |
| Type1 | Ref |  | Ref |  | Ref |  |
| Type2 | 3.42 (2.38–4.92) | <0.001 | 3.94 (2.71-5.74) | <0.001 | 3.94 (2.71–5.73) | <0.001 |
| Type3 | 5.61 (3.93–7.99) | <0.001 | 7.32 (5.06-10.59) | <0.001 | 7.28 (5.03–10.55) | <0.001 |
| Type4 | 11.66 (8.28–16.43) | <0.001 | 13.94 (9.68–20.08) | <0.001 | 13.87 (9.63–19.98) | <0.001 |
| Type5 | 0.75 (0.43–1.30) | 0.310 | 0.65 (0.37–1.14) | 0.137 | 0.65 (0.37–1.13) | 0.131 |
| Type6 | 2.26 (1.53–3.36) | <0.001 | 2.15 (1.43–3.22) | <0.001 | 2.14 (1.42–3.20) | <0.001 |
| Type7 | 4.77 (3.36–6.77) | <0.001 | 5.07 (3.53–7.27) | <0.001 | 5.07 (3.53–7.27) | <0.001 |
| Type8 | 9.46 (6.78–13.20) | <0.001 | 10.29 (7.28–14.54) | <0.001 | 10.30 (7.28–14.59) | <0.001 |
| P for trend | <0.001 | | <0.001 | | <0.001 | |

Model 1: No adjustment.

Model 2: Adjusted for age, sex.

Model 3: Further adjusted for marital status, educational level, TG, FBG, Cr, BUN, smoking and alcohol use.

UHR, uric acid to high-density lipoprotein cholesterol ratio; BRI, body roundness index; OR, odds ratio; CI, confidence interval; CHARLS, China Health and Retirement Longitudinal Study.

Table S7. Weighted logistic regression models for sarcopenia incidence by the joint of the UHR and BRI in the NHANES cohort

| Groups | Model1 | | Model2 | | Model3 | |
| --- | --- | --- | --- | --- | --- | --- |
|  | OR (95%CI) | P-value | OR (95%CI) | P-value | OR (95%CI) | P-value |
| Type1 | Ref |  | Ref |  | Ref |  |
| Type2 | 3.08 (1.46–6.50) | 0.003 | 2.84 (1.34–6.00) | 0.006 | 2.80 (1.32–5.92) | 0.007 |
| Type3 | 15.64 (8.08–30.27) | <0.001 | 14.56 (7.49–28.30) | <0.001 | 12.38(7.39–26.99) | <0.001 |
| Type4 | 46.48 (24.41–88.52) | <0.001 | 43.05 (24.41–80.84) | <0.001 | 40.23(24.61–78.82) | <0.001 |
| Type5 | 0.50 (0.11–2.29) | 0.373 | 0.41 (0.29–1.89) | 0.253 | 0.40 (0.19–1.82) | 0.232 |
| Type6 | 4.97 (2.42–10.18) | <0.001 | 3.56 (1.73–7.34) | <0.001 | 3.43 (1.66–7.06) | <0.001 |
| Type7 | 13.65 (7.09–26.26) | <0.001 | 9.93 (5.14–19.18) | <0.001 | 7.42 (4.88–16.60) | <0.001 |
| Type8 | 39.86 (21.11–75.24) | <0.001 | 33.57 (17.75–63.49) | <0.001 | 30.38 (17.02–56.34) | <0.001 |
| P for trend | <0.001 | | <0.001 | | <0.001 | |

Model 1: No adjustment.

Model 2: Adjusted for age, sex.

Model 3: Further adjusted for marital status, educational level, TG, FBG, Cr, BUN, smoking and alcohol use.

UHR, uric acid to high-density lipoprotein cholesterol ratio; BRI, body roundness index; OR, odds ratio; CI, confidence interval; NHANES, National Health and Nutrition Examination Survey.

Table S8. Interaction between the UHR and BRI on the risk of sarcopenia incidence in the CHARLS cohort

| Interactive indices | Interactive effect(95%CI) |  |  |
| --- | --- | --- | --- |
|  | Model1 | Model2 | Model3 |
| Additive effect |  |  |  |
| RERI | 0.07（-0.15–0.31） | 0.07（-0.16–0.30） | 0.02（-0.20–0.24） |
| AP | 0.04（-0.08–0.16） | 0.04（-0.10–0.18） | -0.01（-0.14–0.13） |
| SI | 1.10（0.81–1.45） | 1.12（0.76–1.49） | 1.02（0.65–1.44） |
| Multiplicative effect | 1.35（1.24–1.51） | 1.31（1.18–1.49） | 1.25（1.20–1.39） |

Model 1: No adjustment.

Model 2: Adjusted for age, sex.

Model 3: Further adjusted for marital status, educational level, TG, FBG, Cr, BUN, smoking and alcohol use.

UHR, uric acid to high-density lipoprotein cholesterol ratio; BRI, body roundness index; RERI, relative excess risk due to interaction; AP, attributable proportion due to interaction; SI, synergy index; CI, confidence interval; CHARLS, China Health and Retirement Longitudinal Study.

Table S9. Interaction between the UHR and BRI on the risk of sarcopenia incidence in the NHANES cohort

| Interactive indices | Interactive effect(95%CI) |  |  |
| --- | --- | --- | --- |
|  | Model1 | Model2 | Model3 |
| Additive effect |  |  |  |
| RERI | 0.11（-0.10–0.24） | 0.09（-0.12–0.34） | 0.05（-0.20–0.40） |
| AP | 0.12（-0.10–0.20） | 0.05（-0.11–0.19） | -0.10（-0.12–0.17） |
| SI | 1.08（0.80–1.45） | 1.05（0.78–1.42） | 0.96（0.76–1.39） |
| Multiplicative effect | 1.28（1.10–1.69） | 1.25（1.08–1.49） | 1.20（1.04–1.37） |

Model 1: No adjustment.

Model 2: Adjusted for age, sex.

Model 3: Further adjusted for marital status, educational level, TG, FBG, Cr, BUN, smoking and alcohol use.

UHR, uric acid to high-density lipoprotein cholesterol ratio; BRI, body roundness index; RERI, relative excess risk due to interaction; AP, attributable proportion due to interaction; SI, synergy index; CI, confidence interval; NHANES, National Health and Nutrition Examination Survey.

Table S10. NRI and IDI values across different models in the CHARLS cohort

| Model | NRI (95%CI) | P value | IDI (95%CI) | P value |
| --- | --- | --- | --- | --- |
| Basic model | Ref | | Ref | |
| Basic model+UHR-BRI | 0.453 (0.221–0.685) | <0.001 | 0.009 (0.002–0.016) | 0.009 |
| Basic model+UHR | 0.268 (0.027–0.509) | 0.041 | 0.003 (0.000–0.006) | 0.047 |
| Basic model+BRI | 0.357 (0.136–0.594) | 0.030 | 0.006 (0.004–0.010) | 0.024 |
| Basic model+BMI | 0.306 (0.147–0.544) | 0.004 | 0.004 (0.001–0.007) | 0.017 |
| Basic model+WC | 0.301 (0.109–0.449) | 0.026 | 0.006 (0.000–0.008) | 0.004 |
| Basic model+CHG | 0.398 (0.229–0.603) | 0.039 | 0.007 (0.002–0.011) | 0.001 |
| Basic model+AIP | 0.313 (0.130–0.548) | 0.027 | 0.003 (0.000–0.007) | 0.006 |
| Basic model+TyG | 0.367 (0.119–0.569) | 0.041 | 0.006 (0.000–0.010) | 0.012 |
| Basic model+UHR-BMI | 0.413 (0.181–0.645) | <0.001 | 0.007 (0.001–0.013) | 0.014 |
| Basic model+UHR-ABSI | 0.428 (0.197–0.659) | <0.001 | 0.008 (0.001–0.015) | 0.011 |
| Basic model+UHR-WC | 0.437 (0.205–0.669) | <0.001 | 0.008 (0.002–0.014) | 0.008 |
| Basic model+UHR-WHtR | 0.419 (0.188–0.650) | <0.001 | 0.007 (0.001–0.013) | 0.013 |
| Basic model+UHR-WWI | 0.275 (0.033–0.517) | 0.039 | 0.003 (0.000–0.006) | 0.044 |

NRI, net reclassification improvement; IDI, integrated discrimination improvement; UHR, uric acid to high-density lipoprotein cholesterol ratio; BRI, body roundness index; BMI, body mass index; WC, waist circumference; WHtR, waist-to-height ratio; ABSI, a body shape index; WWI, waist-to-weight index; TyG, triglyceride–glucose index; AIP, atherogenic index of plasma; CHG, cholesterol, high density lipoprotein, and glucose index; CHARLS, China Health and Retirement Longitudinal Study.

Table S11. NRI and IDI values across different models in the NHANES cohort

| Model | NRI (95%CI) | P value | IDI (95%CI) | P value |
| --- | --- | --- | --- | --- |
| Basic model | Ref | | Ref | |
| Basic model+UHR-BRI | 0.323 (0.163–0.515) | <0.001 | 0.012 (0.006–0.021) | 0.003 |
| Basic model+UHR | 0.213 (0.039–0.335) | 0.002 | 0.008 (0.000–0.014) | 0.004 |
| Basic model+BRI | 0.299 (0.096–0.451) | 0.013 | 0.009 (0.004–0.016) | 0.001 |
| Basic model+BMI | 0.224 (0.049–0.322) | 0.002 | 0.006 (0.000–0.010) | 0.011 |
| Basic model+WC | 0.207 (0.129–0.396) | 0.029 | 0.007 (0.002–0.013) | 0.006 |
| Basic model+CHG | 0.309 (0.199–0.499) | 0.001 | 0.010 (0.004–0.018) | 0.029 |
| Basic model+AIP | 0.217 (0.178–0.394) | 0.002 | 0.006 (0.001–0.015) | 0.008 |
| Basic model+TyG | 0.287 (0.049–0.405) | 0.021 | 0.009 (0.001–0.017) | 0.004 |
| Basic model+UHR-BMI | 0.251 (0.137–0.462) | <0.001 | 0.010 (0.000–0.016) | 0.021 |
| Basic model+UHR-ABSI | 0.303 (0.198–0.431) | <0.001 | 0.006 (0.001–0.015) | 0.035 |
| Basic model+UHR-WC | 0.225 (0.191–0.492) | <0.001 | 0.009 (0.002–0.017) | 0.046 |
| Basic model+UHR-WHtR | 0.343 (0.153–0.452) | <0.001 | 0.011 (0.001–0.019) | 0.008 |
| Basic model+UHR-WWI | 0.283 (0.173–0.482) | 0.037 | 0.008 (0.000–0.014) | 0.029 |

NRI, net reclassification improvement; IDI, integrated discrimination improvement; UHR, uric acid to high-density lipoprotein cholesterol ratio; BRI, body roundness index; BMI, body mass index; WC, waist circumference; WHtR, waist-to-height ratio; ABSI, a body shape index; WWI, waist-to-weight index; TyG, triglyceride–glucose index; AIP, atherogenic index of plasma; CHG, cholesterol, high density lipoprotein, and glucose index; NHANES, National Health and Nutrition Examination Survey.

Table S12. Univariate and multivariate regression analyses of the incidence of sarcopenia in the CHARLS cohort

|  | | Univariate analysis | |  | | Multivariate analysis | |
| --- | --- | --- | --- | --- | --- | --- | --- |
|  | OR | 95%CI | P value |  | OR | 95%CI | P value |
| Age | 1.09 | 1.07-1.10 | <0.001 |  | 1.09 | 1.04-1.14 | 0.012 |
| Gender |  |  |  |  |  |  |  |
| Male | Ref |  |  |  | Ref |  |  |
| Female | 0.75 | 0.66-0.86 | <0.001 |  | 0.95 | 0.78-1.16 | 0.645 |
| Marital Status |  |  |  |  |  |  |  |
| Single | Ref |  |  |  | Ref |  |  |
| Married | 0.37 | 0.32-0.44 | <0.001 |  | 0.79 | 0.66-0.96 | 0.017 |
| Educational Level |  |  |  |  |  |  |  |
| Less than high school | Ref |  |  |  | Ref |  |  |
| High school | 0.92 | 0.83-1.02 | 0.072 |  |  |  |  |
| College or above | 1.14 | 0.97-1.26 | 0.091 |  |  |  |  |
| cuUHR-BRI | 3.35 | 2.74-4.10 | <0.001 |  | 2.78 | 2.17-3.58 | <0.001 |
| Cholesterol | 1.01 | 1.00-1.04 | <0.001 |  | 0.99 | 0.95-1.09 | 0.578 |
| TG | 0.94 | 0.82-1.03 | 0.701 |  |  |  |  |
| FBG | 1.01 | 0.93-1.04 | 0.237 |  |  |  |  |
| LDL | 0.99 | 0.91-1.02 | 0.246 |  |  |  |  |
| BUN | 1.11 | 0.96-1.12 | 0.105 |  |  |  |  |
| CRP | 1.10 | 0.85-1.38 | 0.402 |  |  |  |  |
| Cr | 1.03 | 1.00-1.23 | <0.001 | 0.99 | 0.89-1.15 | 0.205 |  |
| Smoking |  |  |  |  |  |  |  |
| No | Ref |  |  |  | Ref |  |  |
| Yes | 0.72 | 0.63-0.83 | <0.001 |  | 0.76 | 0.63-0.93 | 0.005 |
| Alcohol Use |  |  |  |  |  |  |  |
| No | Ref |  |  |  | Ref |  |  |
| Yes | 0.68 | 0.59-0.79 | <0.001 |  | 0.90 | 0.81-0.98 | 0.025 |
| Obesity |  |  |  |  |  |  |  |
| No | Ref |  |  |  | Ref |  |  |
| Yes | 1.84 | 1.54-2.19 | <0.001 |  | 1.03 | 0.81-1.32 | 0.622 |
| Diabetes |  |  |  |  |  |  |  |
| No | Ref |  |  |  |  |  |  |
| Yes | 0.99 | 0.74-1.33 | 0.947 |  |  |  |  |
| Kidney Diseases |  |  |  |  |  |  |  |
| No | Ref |  |  |  |  |  |  |
| Yes | 0.92 | 0.70-1.19 | 0.546 |  |  |  |  |
| Cancers |  |  |  |  |  |  |  |
| No | Ref |  |  |  |  |  |  |
| Yes | 0.70 | 0.29-1.42 | 0.364 |  |  |  |  |
| Stroke |  |  |  |  |  |  |  |
| No | Ref |  |  |  |  |  |  |
| Yes | 1.44 | 0.95-2.14 | 0.072 |  |  |  |  |
| CVD |  |  |  |  |  |  |  |
| No | Ref |  |  |  |  |  |  |
| Yes | 1.04 | 0.85-1.26 | 0.686 |  |  |  |  |

UHR, uric acid to high-density lipoprotein cholesterol ratio; BRI, body roundness index; TG, triglyceride; FBG, fasting blood glucose; LDL, low density lipoprotein; BUN, blood urea nitrogen; Cr, creatinine; CRP, C-reactive protein; CVD, cardiovascular disease; SD, standard deviation; CHARLS, China Health and Retirement Longitudinal Study.

Table S13. Univariate and multivariate regression analyses of the incidence of sarcopenia in the NHANES cohort

|  | | Univariate analysis | |  | | Multivariate analysis | |
| --- | --- | --- | --- | --- | --- | --- | --- |
|  | OR | 95%CI | P value |  | OR | 95%CI | P value |
| Age | 1.04 | 1.03-1.05 | <0.001 |  | 1.06 | 1.04-1.12 | <0.001 |
| Gender |  |  |  |  |  |  |  |
| Male | Ref |  |  |  | Ref |  |  |
| Female | 1.00 | 0.86-1.16 | 0.987 |  |  |  |  |
| Race |  |  |  |  |  |  |  |
| Mexican American | Ref |  |  |  | Ref |  |  |
| Other Hispanic | 0.65 | 0.61-0.69 | 0.013 |  | 0.74 | 0.70-0.80 | <0.001 |
| Non-Hispanic White | 0.85 | 0.70-1.03 | 0.215 |  | 0.89 | 0.78-1.12 | 0.072 |
| Non-Hispanic Black | 1.17 | 1.03-1.35 | 0.019 |  | 0.81 | 0.67-1.04 | 0.092 |
| Others | 1.36 | 0.95-1.63 | 0.053 |  | 1.14 | 1.02-1.45 | <0.001 |
| Marital Status |  |  |  |  |  |  |  |
| Single | Ref |  |  |  | Ref |  |  |
| Married | 1.20 | 1.03-1.41 | 0.019 |  | 0.96 | 0.84-1.19 | 0.973 |
| Educational Level |  |  |  |  |  |  |  |
| Less than high school | Ref |  |  |  | Ref |  |  |
| High school | 0.89 | 0.63-1.20 | 0.054 |  |  |  |  |
| College or above | 0.94 | 0.87-1.22 | 0.159 |  |  |  |  |
| PIR | 0.85 | 0.81-0.89 | <0.001 |  | 0.95 | 0.89-1.02 | 0.105 |
| UHR-BRI | 3.89 | 3.40-4.42 | <0.001 |  | 4.79 | 3.31-6.97 | <0.001 |
| Cholesterol | 1.05 | 1.02-1.09 | <0.001 |  | 1.04 | 1.01-1.09 | 0.007 |
| TG | 1.03 | 0.91-1.43 | 0.128 |  |  |  |  |
| FBG | 1.01 | 1.00-1.06 | <0.001 |  | 0.98 | 0.88-1.21 | 0.428 |
| LDL | 0.99 | 0.88-1.12 | 0.062 |  |  |  |  |
| WBC | 1.16 | 0.94-1.19 | 0.221 |  |  |  |  |
| RBC | 1.36 | 1.17-1.59 | 0.013 |  | 1.74 | 1.44-2.13 | <0.001 |
| Hb | 1.02 | 0.98-1.08 | 0.249 |  |  |  |  |
| PLT | 1.00 | 1.00-1.01 | <0.001 |  | 1.05 | 0.89-1.17 | 0.062 |
| ALT | 0.99 | 0.90-1.08 | 0.523 |  |  |  |  |
| AST | 1.12 | 1.07-1.42 | 0.041 |  | 1.23 | 1.03-1.59 | <0.001 |
| BUN | 1.09 | 0.92-1.12 | 0.502 |  |  |  |  |
| LDH | 1.01 | 0.96-1.06 | 0.072 |  |  |  |  |
| TBil | 1.25 | 1.11-1.38 | <0.001 |  | 0.87 | 0.75-1.11 | 0.195 |
| Cr | 0.15 | 0.10-0.23 | <0.001 |  | 0.19 | 0.09-0.25 | <0.001 |
| Smoking |  |  |  |  |  |  |  |
| No | Ref |  |  |  | Ref |  |  |
| Yes | 1.22 | 0.53-2.22 | 0.882 |  |  |  |  |
| Alcohol Use |  |  |  |  |  |  |  |
| No | Ref |  |  |  | Ref |  |  |
| Yes | 1.24 | 1.06-1.43 | <0.001 |  | 1.42 | 1.27-1.81 | <0.001 |
| Physical activity |  |  |  |  |  |  |  |
| Insufficient | Ref |  |  |  |  |  |  |
| Sufficient | 1.13 | 0.99-1.28 | 0.562 |  |  |  |  |
| Exceeding | 1.28 | 0.82-1.39 | 0.481 |  |  |  |  |
| Obesity |  |  |  |  |  |  |  |
| No | Ref |  |  |  |  |  |  |
| Yes | 1.68 | 0.89-2.47 | 0.589 |  |  |  |  |
| Diabetes |  |  |  |  |  |  |  |
| No | Ref |  |  |  |  |  |  |
| Yes | 2.70 | 2.17-3.33 | <0.001 |  | 1.20 | 0.87-1.67 | 0.247 |
| Kidney Diseases |  |  |  |  |  |  |  |
| No | Ref |  |  |  |  |  |  |
| Yes | 2.04 | 1.29-3.09 | <0.001 |  | 2.85 | 1.70-4.63 | <0.001 |
| Cancers |  |  |  |  |  |  |  |
| No | Ref |  |  |  |  |  |  |
| Yes | 1.25 | 0.78-1.49 | 0.769 |  |  |  |  |
| Stroke |  |  |  |  |  |  |  |
| No | Ref |  |  |  |  |  |  |
| Yes | 1.94 | 1.17-3.08 | 0.006 |  | 1.02 | 0.75-1.98 | 0.929 |
| CVD |  |  |  |  |  |  |  |
| No | Ref |  |  |  |  |  |  |
| Yes | 2.23 | 1.64-2.98 | <0.001 |  | 1.40 | 0.89-2.15 | 0.133 |

UHR, uric acid to high-density lipoprotein cholesterol ratio; BRI, body roundness index ;PIR, poverty income ratio; TG, triglyceride; FBG, fasting blood glucose; LDL, low density lipoprotein; WBC, white blood cell; RBC, red blood cell; Hb, hemoglobin; PLT, platelet; ALT, alanine aminotransferase; AST, aspartate aminotransferase; BUN, blood urea nitrogen; LDH, lactate dehydrogenase; TBil, total bilirubin; Cr, creatinine; CVD, cardiovascular disease; NHANES, National Health and Nutrition Examination Survey.

Table S14. Association between UHR-BRI and the risk of sarcopenia without imputation in the CHARLS cohort

| Exposure | Model1 | | Model2 | | Model3 | |
| --- | --- | --- | --- | --- | --- | --- |
|  | OR (95%CI) | P-value | OR (95%CI) | P-value | OR (95%CI) | P-value |
| Per 1-SD increment | 3.32 (2.68-4.26) | <0.001 | 3.27 (2.53-4.11) | <0.001 | 3.26 (2.47-3.99) | <0.001 |
| Groups |  |  |  |  |  |  |
| cuUHR-BRI Q1 | Ref |  | Ref |  | Ref |  |
| cuUHR-BRI Q2 | 1.88 (1.18-2.43) | <0.001 | 1.65 (1.12-2.07) | <0.001 | 1.54 (0.98-1.96) | 0.062 |
| cuUHR-BRI Q3 | 2.46 (1.59-3.66) | <0.001 | 2.29 (1.28-3.12) | <0.001 | 2.07 (1.02-2.99) | <0.001 |
| cuUHR-BRI Q4 | 3.55 (2.31-4.76) | <0.001 | 3.31 (2.45-4.20) | <0.001 | 3.19 (2.26-3.85) | <0.001 |
| P for trend | <0.001 |  | <0.001 |  | <0.001 |  |

Model 1: No adjustment.

Model 2: Adjusted for age, sex.

Model 3: Further adjusted for marital status, educational level, TG, FBG, Cr, BUN, smoking and alcohol use.

UHR, uric acid to high-density lipoprotein cholesterol ratio; BRI, body roundness index; OR, odds ratio; CI, confidence interval; CHARLS, China Health and Retirement Longitudinal Study.

Table S15. Association between UHR-BRI and the risk of sarcopenia without imputation in the NHANES cohort

| Exposure | Model1 | | Model2 | | Model3 | |
| --- | --- | --- | --- | --- | --- | --- |
|  | OR (95%CI) | P-value | OR (95%CI) | P-value | OR (95%CI) | P-value |
| Per 1-SD increment | 3.79 (3.18-4.44) | <0.001 | 3.70 (3.11-4.42) | <0.001 | 3.45 (2.91-4.10) | <0.001 |
| Groups |  |  |  |  |  |  |
| UHR-BRI Q1 | Ref |  | Ref |  | Ref |  |
| UHR-BRI Q2 | 3.42 (2.26-5.33) | 0.001 | 3.34 (2.24-5.21) | <0.001 | 3.33 (2.17-5.17) | <0.001 |
| UHR-BRI Q3 | 6.31 (4.26-9.69) | <0.001 | 6.07 (4.09-9.32) | 0.006 | 5.81 (3.93-8.88) | 0.004 |
| UHR-BRI Q4 | 11.77 (8.02-17.9) | 0.022 | 11.35 (7.72-17.31) | 0.016 | 10.39 (7.13-15.71) | <0.001 |
| P for trend | <0.001 |  | <0.001 |  | <0.001 |  |

Model 1: No adjustment.

Model 2: Adjusted for age, sex.

Model 3: Further adjusted for marital status, educational level, TG, FBG, Cr, BUN, smoking and alcohol use.

UHR, uric acid to high-density lipoprotein cholesterol ratio; BRI, body roundness index; OR, odds ratio; CI, confidence interval; NHANES, National Health and Nutrition Examination Survey.

Table S16. The proportion of missing covariates

| Cohort | Variable | Number | Proportion |
| --- | --- | --- | --- |
| CHARLS |  |  |  |
|  | Cr | 852 | 9.98 |
|  | BUN | 842 | 9.86 |
|  | CRP | 716 | 8.39 |
|  | LDL | 518 | 6.07 |
|  | Alcohol Use | 406 | 4.76 |
|  | Education Level | 377 | 4.42 |
|  | Marital Status | 312 | 3.65 |
|  | Smoking | 285 | 3.34 |
| NHANES |  |  |  |
|  | LDL | 827 | 9.72 |
|  | Cr | 805 | 9.46 |
|  | AST | 730 | 8.58 |
|  | ALT | 819 | 9.62 |
|  | BUN | 793 | 9.32 |
|  | Education Level | 380 | 4.47 |
|  | Alcohol Use | 445 | 5.23 |
|  | Smoking | 426 | 5.01 |
|  | TBil | 806 | 9.47 |
|  | LDH | 790 | 9.28 |
|  | Marital Status | 399 | 4.69 |
|  | WBC | 584 | 6.86 |
|  | Hb | 569 | 6.69 |
|  | PLT | 529 | 6.22 |
|  | RBC | 467 | 5.49 |
|  | PIR | 376 | 4.42 |

PIR, poverty income ratio; LDL, low density lipoprotein; WBC, white blood cell; RBC, red blood cell; Hb, hemoglobin; PLT, platelet; ALT, alanine aminotransferase; AST, aspartate aminotransferase; BUN, blood urea nitrogen; LDH, lactate dehydrogenase; TBil, total bilirubin; Cr, creatinine; CRP, C-reactive protein; NHANES, National Health and Nutrition Examination Survey; CHARLS, China Health and Retirement Longitudinal Study.

**Details of Multiple Imputation by Chained Equations:**

To minimize potential bias from complete-case exclusion, multiple imputation by chained equations (MICE) was performed using the mice package in R. A total of m = 5 imputed datasets were generated, with 20 iterations per imputation. Imputation was conducted under the missing-at-random (MAR) assumption, and continuous variables were imputed using predictive mean matching (PMM), binary and ordered categorical variables were imputed using logistic regression and ordered logistic regression, respectively.

The proportions of missing values for key covariates are summarized in Table S16. Convergence was evaluated by analyzing trace plots depicting the mean and standard deviation of imputed values across all imputation chains, which demonstrated sufficient convergence and favorable mixing for all variables (Figure S14-S17). Pooled estimates across imputed datasets were obtained using Rubin’s rules.

As a sensitivity analysis, results from complete-case and multiple imputation were compared for the primary outcome model, the two approaches remained highly consistent in both cohorts (Table2, S14,15).

Fig S1. Flowchart of the inclusion and exclusion process. NHANES, National Health and Nutrition Examination Survey; CHARLS, China Health and Retirement Longitudinal Study; UHR, uric acid to high-density lipoprotein cholesterol ratio; BRI, body roundness index; OBS, oxidative balance score.

Fig S2. ROC curves of UHR-BRI and other indices for sarcopenia. (A) In the CHARLS cohort, (B) In the NHANES cohort. UHR, uric acid to high-density lipoprotein cholesterol ratio; BRI, body roundness index; BMI, body mass index; WC, waist circumference; TyG, triglyceride–glucose index; AIP, atherogenic index of plasma; CHG, cholesterol, high density lipoprotein, and glucose index; NHANES, National Health and Nutrition Examination Survey; CHARLS, China Health and Retirement Longitudinal Study.

Fig S3. ROC curves of UHR-BRI and other indices for sarcopenia comorbidity in the NHANES cohort. (A) Sarcopenic obesity, (B) S-KD comorbidity, (C) S-DM comorbidity, (D) S-CVD comorbidity, (E) S-stroke comorbidity, (F) S-cancer comorbidity. UHR, uric acid to high-density lipoprotein cholesterol ratio; BRI, body roundness index; BMI, body mass index; WC, waist circumference; WHtR, waist-to-height ratio; ABSI, a body shape index; WWI, waist-to-weight index; ROC, receiver operating characteristic; S-KD, sarcopenia-kidney disease; S-DM, sarcopenia- diabetes mellitus, S-CVD, sarcopenia-cardiovascular disease; S-stroke, sarcopenia-stroke; S-cancer, sarcopenia-cancer; NHANES, National Health and Nutrition Examination Survey.

Fig S4. AUC values of UHR-BRI and other indices for sarcopenia comorbidity. (A) CHARLS cohort, (B) NHANES cohort. UHR, uric acid to high-density lipoprotein cholesterol ratio; BMI, body mass index; WC, waist circumference; TyG, triglyceride–glucose index; AIP, atherogenic index of plasma; CHG, cholesterol, high density lipoprotein, and glucose index; S-KD, sarcopenia-kidney disease; S-DM, sarcopenia- diabetes mellitus, S-CVD, sarcopenia-cardiovascular disease; S-stroke, sarcopenia-stroke; S-cancer, sarcopenia-cancer; NHANES, National Health and Nutrition Examination Survey CHARLS, China Health and Retirement Longitudinal Study.

Fig S5. GBTM analysis of UHR, BRI across study waves. (A) Scatter plots of individual observed UHR values across study waves, (B) Fitted longitudinal trajectory curves of UHR, (C)Bar charts displaying the incidence of sarcopenia stratified by trajectory group of UHR, (D) Scatter plots of individual observed BRI values across study waves, (E) Fitted longitudinal trajectory curves of BRI, (F)Bar charts displaying the incidence of sarcopenia stratified by trajectory group of BRI. GBTM, group-based trajectory modeling; UHR, uric acid to high-density lipoprotein cholesterol ratio; BRI, body roundness index.

Fig S6. Boruta feature importance over iterations. (A) In the NHANES cohort, (B) In the CHARLS cohort. NHANES, National Health and Nutrition Examination Survey; CHARLS, China Health and Retirement Longitudinal Study.

Fig S7. Correlation heatmap assessing multicollinearity among key predictors. (A) In the NHANES cohort, (B) In the CHARLS cohort. UHR, uric acid to high-density lipoprotein cholesterol ratio; BRI, body roundness index; RBC, red blood cell; AST, aspartate aminotransferase; TC, total cholesterol; Cr, creatinine; NHANES, National Health and Nutrition Examination Survey; CHARLS, China Health and Retirement Longitudinal Study.

Fig S8. 10-Fold Cross-Validation of the XGB Model for sarcopenia prediction. (A) In the NHANES cohort, (B) In the CHARLS cohort. XGB, extreme gradient boosting; NHANES, National Health and Nutrition Examination Survey; CHARLS, China Health and Retirement Longitudinal Study.

Fig S9. DCA of the XGB Model for sarcopenia prediction. (A) In the NHANES cohort, (B) In the CHARLS cohort. XGB, extreme gradient boosting; DCA, decision curve analysis; NHANES, National Health and Nutrition Examination Survey; CHARLS, China Health and Retirement Longitudinal Study.

Fig S10. Calibration curve of the XGB Model for sarcopenia prediction. (A) In the NHANES cohort, (B) In the CHARLS cohort. XGB, extreme gradient boosting; NHANES, National Health and Nutrition Examination Survey; CHARLS, China Health and Retirement Longitudinal Study.

Fig S11. The SHAP values of each variable in the XGB model for predicting the risk of sarcopenia incidence in the NHANES cohort. UHR, uric acid to high-density lipoprotein cholesterol ratio; BRI, body roundness index; RBC, red blood cell; AST, aspartate aminotransferase; Cr, creatinine; TC, total cholesterol; XGB, extreme gradient boosting; SHAP, shapley additive explanations; NHANES, National Health and Nutrition Examination Survey.

Fig S12. The SHAP values of each variable in the XGB model for predicting the risk of sarcopenia incidence in the CHARLS cohort. UHR, uric acid to high-density lipoprotein cholesterol ratio; BRI, body roundness index; CHARLS, China Health and Retirement Longitudinal Study.

Fig S13. Subgroup analyses of UHR‑BRI with sarcopenia and sarcopenia comorbidities in the NHANES cohort. (A) Sarcopenia, (B) Sarcopenic obesity, (C) Sarcopenia-diabetes comorbidity, (D) Sarcopenia-cardiovascular disease comorbidity. OR, odds ratio; CI, confidence interval; CVD, cardiovascular disease; NHANES, National Health and Nutrition Examination Survey.

Fig S14. Trace plots of mean values of covariates in the CHARLS cohort. LDL, low density lipoprotein; BUN, blood urea nitrogen; Cr, creatinine; CRP, C-reactive protein; CHARLS, China Health and Retirement Longitudinal Study.

Fig S15. Trace plots of standard deviation values of covariates in the CHARLS cohort. LDL, low density lipoprotein; BUN, blood urea nitrogen; Cr, creatinine; CRP, C-reactive protein; CHARLS, China Health and Retirement Longitudinal Study.

Fig S16. Trace plots of mean values of covariates in the NHANES cohort. PIR, poverty income ratio; LDL, low density lipoprotein; WBC, white blood cell; RBC, red blood cell; Hb, hemoglobin; PLT, platelet; ALT, alanine aminotransferase; AST, aspartate aminotransferase; BUN, blood urea nitrogen; LDH, lactate dehydrogenase; TBil, total bilirubin; Cr, creatinine; NHANES, National Health and Nutrition Examination Survey.

Fig S17. Trace plots of standard deviation values of covariates in the NHANES cohort. PIR, poverty income ratio; LDL, low density lipoprotein; WBC, white blood cell; RBC, red blood cell; Hb, hemoglobin; PLT, platelet; ALT, alanine aminotransferase; AST, aspartate aminotransferase; BUN, blood urea nitrogen; LDH, lactate dehydrogenase; TBil, total bilirubin; Cr, creatinine; NHANES, National Health and Nutrition Examination Survey.
